# Anti-Zika virus activity and chemical characterization by ultra-high performance liquid chromatography (UPLC-DAD-UV-MS) of ethanol extracts in *Tecoma* species

**DOI:** 10.1186/s12906-020-03040-0

**Published:** 2020-08-07

**Authors:** Adriana Cotta Cardoso Reis, Breno Mello Silva, Hélia Maria Marques de Moura, Guilherme Rocha Pereira, Geraldo Célio Brandão

**Affiliations:** 1 Pharmacy Department, School of Pharmacy, Federal University of Ouro Preto, Campus Morro do Cruzeiro, Ouro Preto, Minas Gerais 35400-000 Brazil; 2 Department of Biological Sciences, ICEB, Federal University of Ouro Preto, Campus Morro do Cruzeiro, Ouro Preto, Minas Gerais Brazil; 3 Department of Physics and Chemistry, Institute of Exact Sciences and IT (ICEI), Catholic Pontifical University of Minas Gerais, PUC Minas, Belo Horizonte, Minas Gerais Brazil

**Keywords:** *Zika virus*, Antiviral activity, *Tecoma castaneifolia*, *Tecoma garrocha*, *Tecoma stans*, Phenylethanoid glycoside, Flavonoids, Lignans

## Abstract

**Background:**

Plant species from the genus *Tecoma* are found in tropical and subtropical regions around the world. Some of them are grown as ornamental plants and others can be used as medicinal plants. In the present study, ethanolic extracts from trunks and leaves of *Tecoma* species were tested in vitro using assays against the *Zika virus.*

**Methods:**

There was a total of 8 extracts obtained from different anatomical parts of three *Tecoma* species. The *Tecoma castaneifolia*, *T. garrocha*, *T. stans* var. *angustata* and *T. stans* var. *stans* were prepared by percolation with ethanol. The antiviral activity was assayed in vitro against the *Zika virus* by the MTT colorimetric method (*n* = 3). The UPLC-DAD-MS analysis of ethanolic extracts was performed from all the studied species. The biofractionation of *T. stans* var. *stans* trunk extract using different separation techniques led to the isolation of crenatoside compound.

**Results:**

Ethanolic extract from *Tecoma* species leaves were more active against the *Zika virus* (EC_50_ 149.90 to 61.25 μg/mL) when compared to the trunk extracts tested (EC_50_ 131.0 to 66.79 μg/mL and two were not active). The ethyl acetate and aqueous fractions obtained from *T. stans* var. *stans* trunk were active against the *Zika virus* with EC_50_ values of 149.90 and 78.98 μg/mL, respectively. Crenatoside is a phenylethanoid glycoside isolated from the ethyl acetate of *T. stans* var. *stans* trunk extract. This compound was tested and exhibited EC_50_ 34.78 μM (21.64 μg/mL), thus demonstrating a better result than the original ethanolic extracts as well as others extracts of *Tecoma* species, and it was more active than the positive control, ribavirin (386.84 μM). Furthermore, its selectivity index was at least 2.5 times higher than the tested ethanolic extracts and 11.1 times more potent than ribavirin.

**Conclusion:**

The *Tecoma* species demonstrated interesting in vitro activity against the *Zika virus*. The crenatoside, phenylethanoid glycoside that was for the first time isolated from *Tecoma stans* var. *stans*, exhibited a potent and relevant anti-*Zika virus* activity, being more active than ribavirin (positive control). The data show that crenatoside, was a promising compound with in vitro antiviral activity against the *Zika virus*.

## Background

The *Zika virus* (ZIKV) is an infectious mosquito-borne flavivirus. It was first reported in 1947, when monkeys from the Zika Forest in Uganda were infected. Later, the first human casualties were described in Nigeria in 1954. It could be transmitted by different species of *Aedes* mosquitoes in tropical and subtropical areas. Several cases were described in the Americans, Asia and Africa from 1960s to 1980s. At a later date, there were outbreaks reported in different countries in the pacific. In 2015, Brazil reported the first cases of the *Zika virus* in Rio Grande do Norte and Bahia [1].

The infection of the *Zika virus* can be asymptomatic or it may only present the classic symptoms of dengue. Usually, the symptoms start from 3 to 12 days after the mosquito bite. There are ongoing studies of complications caused by the *Zika virus* during pregnancy*.* Recently, the *Zika virus* infection has been linked to cases of microcephaly and pregnancy complications even including fetal loss. Both adults and young people infected can also develop myelitis, neuropathy and Guillain-Barré syndrome, an autoimmune disease in which the immune system attacks the nervous system causing nerve inflammation and muscular weakness [2].

The antivirus therapies play an important role in the research field since viral infections remain a major cause of death around the world. These infections can be controlled, through either preventive prophylactic therapeutic measures (vaccines) or healing drugs. In this context, plants are the source of several bioactive molecules with antiviral activity [3].

Bignoniaceae is a large family of plants that can be found mainly in Central and South America, Africa and Asia. Different metabolites are found, such as terpenoids, naphthoquinones, flavonoids, phenolic compounds among other groups [4]. Some of these compounds presented have antiviral activity [3]. A previous work has shown that Bignoniaceae species are promising sources of antiviral compounds [5–10], including phenylethanoids [6, 8], which justifies the studies of the antiviral activity in the species of the genus *Tecoma*, an important group of plants belonging to this botany family. The data obtained in the present study using chromatographic analyzes combined with hyphenated spectrometric techniques showed that the ethanolic extracts of the species under study were rich in phenylethanoids.

A recent study of extracts from *Fridericia formosa* were promising since some xanthones were isolated and identified with activity against *Herpes virus*, *Vaccinia virus* and the *Dengue virus* 2 [5]. Previously, Kernan et al., [6] isolated five phenylpropanoid glycosides from the *Markhamia lutea* species with potent in vitro activity against *Respiratory Syncytial virus*. There was another relevant discovery during the 1980s, which were antiviral activity reports of lapachol and some of its derivatives, a common naphthoquinone isolated from different Bignoniaceae species [7].

The shrubs and small native trees of the genus *Tecoma* Juss., with mostly 14 species, are found in tropical regions as described for this family [11]. Some of them produce exuberant flowers, which are used for ornamental purpose in numerous countries, including Brazil [11, 12]. Folk tradition reports the use of these plants as anti-syphilitic, tonic, vermifuge, and diuretic. In Mexico, the extract of *Tecoma stans* is employed in the control of diabetes. Additional studies of *Tecoma* plants report isolation and identification of abundant monoterpenic alkaloids. In addition, other secondary metabolites including lapachol, ursolic acid and apigenin were found [13].

The present work shows the anti-*Zika virus* activity from ethanolic extracts of *Tecoma* species. Further, phytochemical studies of ethanolic extracts were accomplished using ultra performance liquid chromatography with a coupled mass spectrometer (LCMS). Also, the chromatographic fractionation of *T. stans* trunk extract was performed.

## Methods

### Plant materials

The following plant species [*T. casneifolia* (BHCB 169768)*, T. garrocha* (BHCB 169765)*, T. stans* var. *angustata* (BHCB 162001) and *T. stans* var. *stans* (BHCB 130095)] were collected in Minas Gerais, Brazil, and a taxonomic determination was made by the botanist Dr. J. R. Stehman, from the Botanical Department at the Institute of Biological Sciences, UFMG, in Belo Horizonte city, Brazil. A voucher of each species was deposited at the BHCB/UFMG herbarium.

### Preparation of extracts

The different parts of the plants (leaves and trunks) were separated and dried in a forced ventilation oven at 40 °C. Then, the plant material was ground in a knife mill and extracted with 96% ethanol at room temperature. The solvent was removed in a Büchi Rotary Evaporator under the reduced pressure and at the control temperature of 50 °C, leaving dark residues which were kept in a vacuum desiccator until constant weight. The extractive yields of ethanol extracts obtained by cold percolation with ethanol are shown in Table 1.

**Table 1:** Extractive yield of plant material from species of the genus *Tecoma*

**Specie**		**Plant material mass (g)**	**Extract mass (g)**	**Yields (%)**
***Tecoma castaneifolia***	Trunk	80.0	3.0	3.8
	Leaves	349.5	30.3	8.7
***Tecoma garrocha***	Trunk	55.0	6.2	11.3
	Leaves	19.0	2.1	11.1
***Tecoma stans*var.*angustata***	Trunk	152.5	10.7	7.0
	Leaves	83.0	5.9	7.1
***Tecoma stans*var.*stans***	Trunk	463.0	54.5	11.8
	Leaves	136.0	9.9	7.3

### Apparatus

Analyses of LC-DAD-MS and LC-ESI-MS/MS were performed using a UPLC Acquity (Waters) ion trap mass spectrometer in the following conditions: positive and negative ion mode; capillary voltage, 3500 V; capillary temperature, 320 °C; source voltage, 5 kV; vaporizer temperature, 320 °C; corona needle current, 5 mA; and sheath gas, nitrogen, 27 psi. The analyses were conducted in the full scan mode (100–2000 Da). The ESI-MS^2^ analyses were additionally performed in a UPLC Acquity (Waters) with argon as the collision gas and the collision energy was set at 30 eV. Chromatographic separation was done on ACQUITY UPLC HSS column (1.7 μm, 50 × 2 mm i.d.) (Waters). The mobile phase consisted of water 0.1% formic acid (solvent A) and acetonitrile 0.1% formic acid (solvent B). The elution protocol was 0–11 min, linear gradient from 5 to 95% B. The flow rate was 0.3 mL min − 1, and the sample injection volume was 4.0 μL. The UV spectra was registered from 190 to 450 nm. The mass spectrometry analysis was performed by Waters ACQUITY® TQD equipped with a quadrupole instrument fitted with an electrospray source in the positive and negative ESI mode. The ion spray voltage was: − 4 kV; orifice voltage: − 60 V.

Analyzes of the ^13^C and ^1^H NMR spectra were used to determine the structure of the isolated compound crenatoside. These analyzes were obtained in the Multi-user Molecule Characterization Laboratory at the Pharmacy School (UFOP) in Ouro Preto, Brazil. A Bruker Ascend™ 400 equipment was used to obtain the spectra. The solvents used were DMSO_*d6*_ and MeOD with TMS as internal standard. The chemical shifts are given as δ (ppm).

### Bioguided fractionation of the trunk extract from *Tecoma stans* var. *stans*

**Fig. 1:**
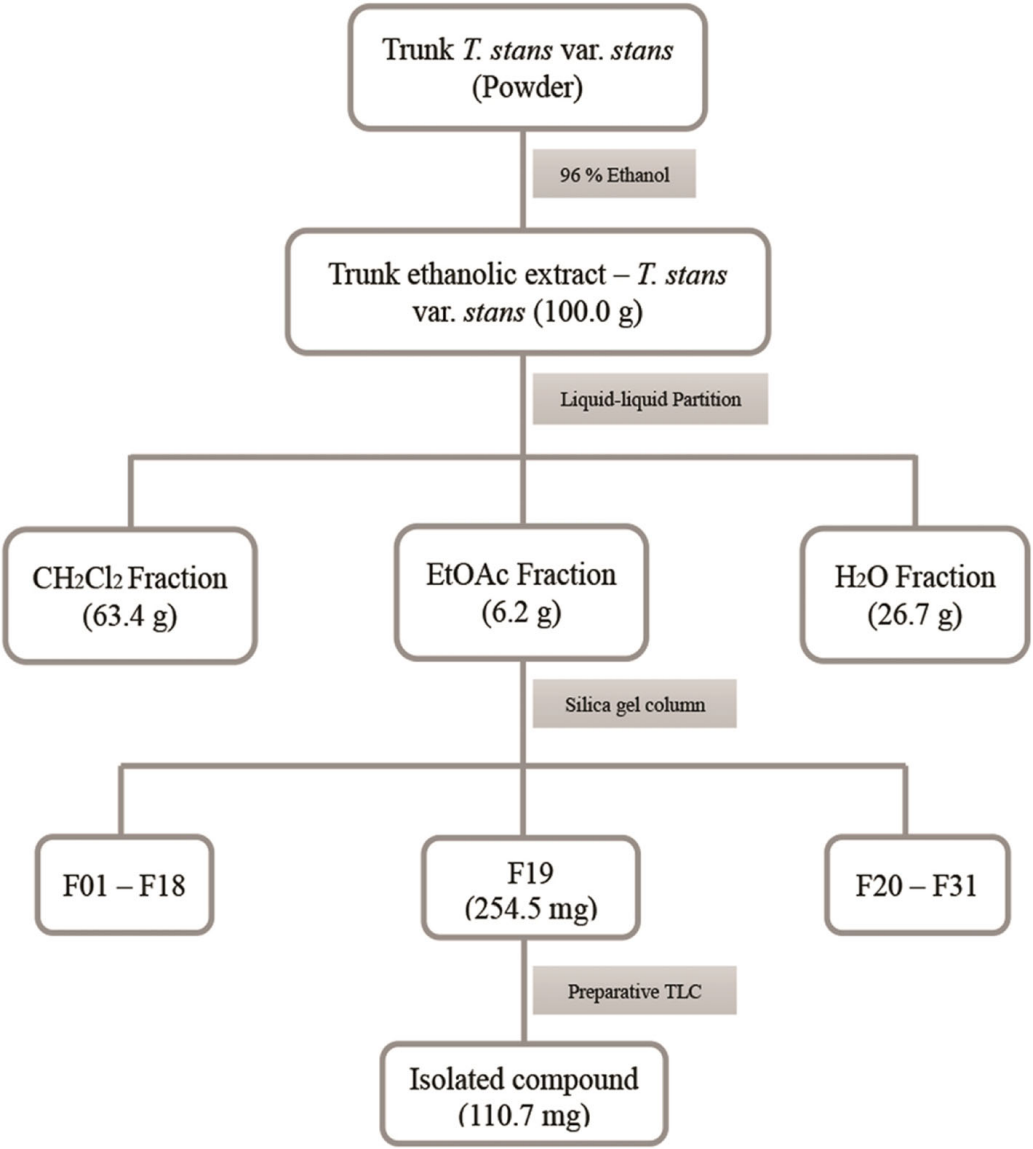
Fractionation of the ethanolic extract from *T. stans* var. *stans* trunk

A portion of the trunk ethanol extract from *T. stans* var. *stans* (50.0 g) was dissolved in a methanol-water (6:4) solution. Then, it was fractionated by liquid-liquid partition with dichloromethane (CH_2_Cl_2_) and ethyl acetate (EtOAc), sequentially. The corresponding *T. stans* dichloromethane trunk extract (13.4 g), *T. stans* ethyl acetate trunk extract (19.6 g) and *T. stans* aqueous trunk extract (12.3 g) fractions were obtained (Fig. 1).

These three fractions were tested in vitro against *Zika virus* and *T. stans* ethyl acetate and the aqueous trunk extract fractions were active. The *T. stans* ethyl acetate was chosen to be fractionated. Hence, a portion of the EtOAc fraction (8.0 g) obtained from the liquid-liquid partition (from ethanolic extract) was also fractionated on a silica gel column (silica gel 60–230-400 mesh-Merck®). It was used by increasing the polarity of the solvents in the following order: CH_2_Cl_2,_ CH_2_Cl_2_/EtOAc (1:1), EtOAc, MeOH. Finally, 282 fractions were obtained from this purification. Similar fractions were united according to their TLC chromatographic profiles, thus obtaining 31 combined fractions. One of these combined fractions, containing 254.5 mg was rechromatographed on preparative TLC (silica gel 60 F_254_-Merck®; 20 × 20 cm, layer thickness 1.0 mm) using CH_2_Cl_2_/EtOAc (8:2) as the mobile phase. The result of this new purification was an isolated white compound that yielded 110.7 mg (Fig. 1) and was identified as crenatoside.

### Virus and cell line

The kidney cells of the African green monkey *Cercopthecus aeothiops* (Vero cell line ATCC® CCL-81™) was used in the assays. The cells were cultivated in complete cell medium consisting of Dulbecco’s modified Eagle’s medium (DMEM, Cultilab, Campinas, SP, Brazil), supplemented with 5% fetal bovine serum, 50 μg/mL gentamicin, 100 U/mL penicillin and 5 μg/mL amphotericin B [8]. The cells were grown in 37 °C in a humidified atmosphere containing 5% CO_2_ and harvested in log-phase for experimental use. The Zika virus strain was donated by Dra. E. Kroon (UFMG, Belo Horizonte, Brazil). The virus was titrated by TCID_50_ in Vero cells, as established by Rodriguez et al.*,* in 1990 [14] and the titers were 1.0 × 10^7^ TCID_50_ / mL.

### Cytotoxicity assay

The MRC-5 (normal human lung fibroblast cell, ATCC® CCL-117™) and Vero (normal African green monkey kidney cells, ATCC® CCL-81™) cell lines were exposed to different concentrations of extracts/fractions/compounds for 48 h and 72 h, respectively [8]. After incubation, cell viability was assessed by the 3-(4,5-dimethylthiazol-2-yl)-2,5-diphenyltetrazolium bromide (MTT, Sigma Aldrich) assay at a concentration of 2 mg/mL in PBS [14, 15]. Each sample was assayed in three replicates in concentrations ranging from 200 to 0.125 μg/mL for crude ethanol extracts, for the isolated compound the concentrations ranged from 100 to 0.781 μg/mL, and for the ribavirin (positive control), the concentrations ranged from 400 to 6.25 μg/mL. The cytotoxicity of each sample was expressed as CC_50_, i.e. and the sample concentration inhibited cell growth by 50% [8].

### Antiviral MTT assays

The antiviral activity measured by the effective dose of 50% (EC_50_) ethanolic extract of *Tecoma* species, fractions from *T. stans* and isolated compound was evaluated by the MTT assay [16]. The Vero cell monolayer (2.0 × 10^4^ cells per well) was infected by viral suspensions with titers of 1.0 × 10^7^ TCID_50_/mL, (MOI = 1.0), and the *Zika virus*. Dilutions of the compounds and ethanolic extract in non-cytotoxic concentrations were added to the wells after viral infection. The plates were incubated at 37 °C in humidified 5% CO_2_ atmosphere for a period of 72 h [8]. The experiments were carried out with eight different concentrations within the non-cytotoxic range of the samples. Ribavirin, an antiviral drug known to be active against the *Zika virus* [17], was used as a positive control to demonstrate that the proposed assay is reliable in determining anti-*Zika virus* activity, in addition to being a comparison parameter with the samples tested in relation to the evaluation of the antiviral activity. The DMSO is a solvent used to dilute the samples, and it was used to demonstrate that DMSO has no anti-*Zika virus* activity (negative control). The 50% inhibitor concentration of the viral effect (EC_50_) for compounds and ethanolic extract were calculated from concentration-effect-curves after the nonlinear regression analysis [8]. The selective index (SI) is defined as CC_50_ over EC_50_ in Vero cell line. The SI is considered interesting for values higher than two [18, 19].

### Statistical analyses

The statistical calculations of the cytotoxic and antiviral MTT assays were performed with the GraphPad prism 5.0 software package (Statistica). The results are expressed as the mean ± S.D. of 4 in independent experiments. Student’s t-test was used for statistical analyses; *P* values > 0.05 were considered to be significant.

### In vitro Cytopathic effect inhibition assay

In order to confirm the anti-ZIKV activity observed in the antiviral MTT assay, the Vero cell monolayer (9.5 × 10^6^ cells per well) was infected by viral suspensions with titers of 1.0 × 10^7^ TCID_50_/mL, (MOI = 1), and the *Zika virus*, during 1 h for the viral adsorption. Afterwards, the viral suspension was removed and the wells were washed with PBS. Then, the wells were treated with the active concentration of the compound (25 μg/mL) and ethanolic extracts of *Tecoma* species: *T. casneifolia* trunk and leaves (50 μg/mL)*, T. garrocha* trunk and leaves (200 μg/mL)*, T. stans* var. *angustata* leaves (50 μg/mL) and *T. stans* var. *stans* leaves (100 μg/mL). The plates were incubated at 37 °C in a humidified 5% CO_2_ atmosphere and photographed 24 h post-infection [20].

## Results

### UPLC analysis and identification of compounds from Tecoma species

The screening, identification, and further confirmation of several components in the studied extracts were performed by UPLC–DAD-MS. This study used a spectrometric method to provide molecular mass ions and characteristic fragment ions. There were eight isolated compounds such as rutin, verbascoside, apigenin, paulownin, paulownin acetate, sesamin, olivil and cycloolivil (Lab Collection), which were used as standards. These standard compounds were used for optimization during separation of phenolic compounds in the UPLC, as well as for the ionization and fragmentation using ESI MS^2^.

**Fig. 2:**
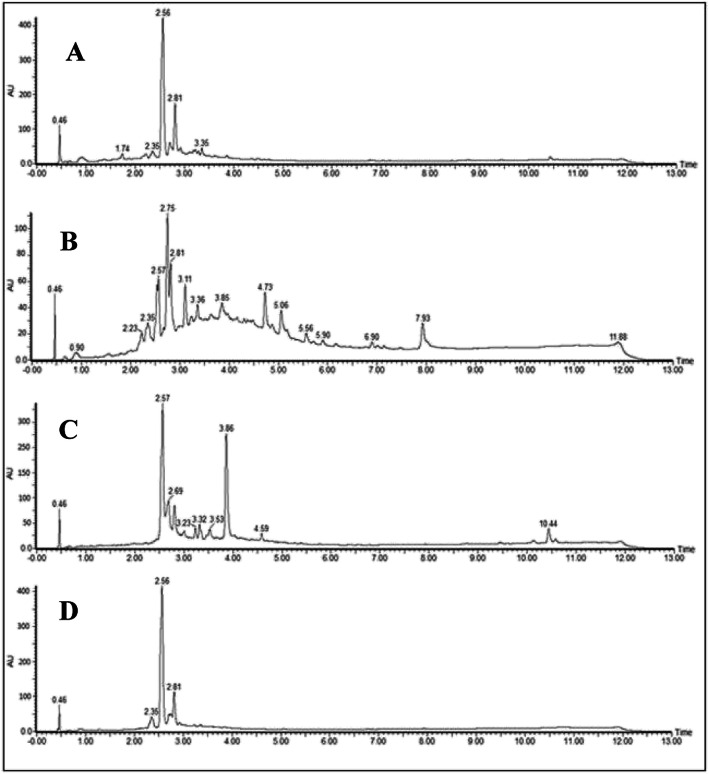
Chromatographic profile of ethanolic extracts of leaves (**a**), trunk (**b**) of *T. castaneifolia* and leaves (**c**), trunk (**d**) of *T. garrocha*

**Fig. 3:**
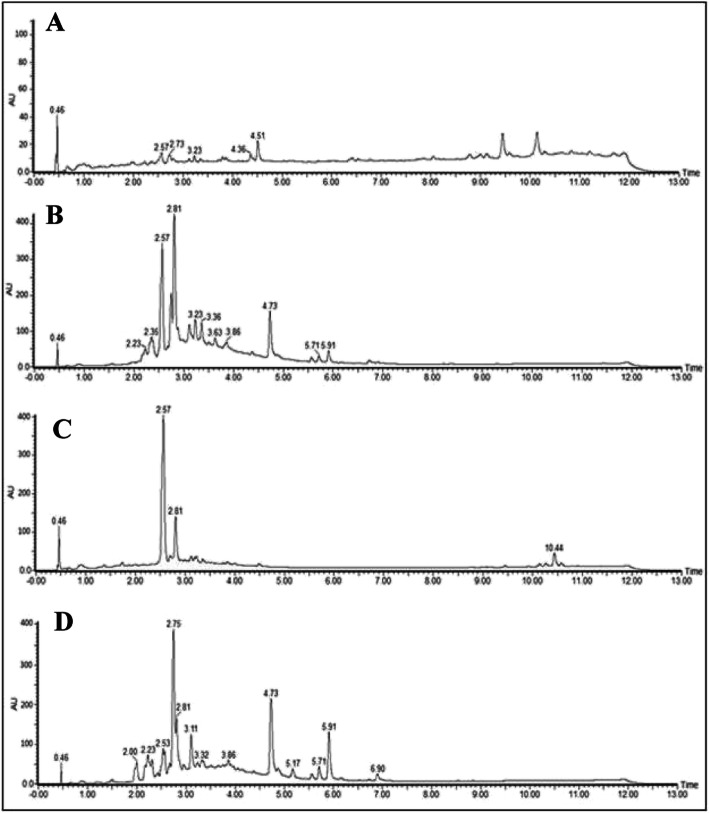
Chromatographic profile of ethanolic extracts of leaves (**a**), trunk (**b**) of *T. stans* var. *angustata* and leaves (**c**), trunk (**d**) of *T. stans* var. *stans*

The UPLC-DAD fingerprints for the crude ethanolic extracts from *Tecoma* species are presented in Figs. 2 and 3. The sensitivity was higher when chromatograms were acquired in negative-ion mode mass spectra and it is shown in the Supplementary Material (Fig. 1S to 8S). The positive-ion mode only provided a few peaks. A tentative identification of plant components was performed by detailed fragmentation studies. The obtained spectra were also compared with published data from the literature.

The UPLC-DAD-MS analysis allowed for the identification of phenylethanoids glycoside as one of the main constituents in all the extracts as inferred from their UV spectra which were registered online. Verbascoside and isoverbascoside were the most common detected phenylethanoids. Isoverbascoside is present in all analyzed extracts, exhibiting a retention time of approximately 2.57 min (Figs. 2 and 3, Table 2). The verbascoside was not detected in the leaves extract of *T. stans* var. *angustata*.

**Table 2:** Phenolic moieties identified in ethanolic extract of leaves and trunk of *Tecoma* species

Compounds	RT (min)	UV (nm)	[M + H]^+^ (m/z)	[M-H]^−^ (m/z)	Fragments (m/z)
***T. stans var stans*(leaves)**					
Isoverbascoside	2.57	330.6	625.75	623.66	623.45, 461.17, 315.11, 297.21, 161.96
Verbascoside	2.81	329.4	625.68	623.65	623.23, 461.21, 315.14, 297.15, 161.89
***T. stans var stans*(trunk)**					
Phenylpropanoid glycoside	2.21	324.5	^a^-	639.60	639.28, 621.16, 469.35, 459.40, 323.45, 313.19, 179.0, 161.2, 151.25
Olivil	2.23	283.4	–	375.51	^b^359.31; 341.35; 323.25; 311.29
Isoleucosceptoside A	2.43	285.3, 324.5	–	637.21	637.21, 475.07, 329.17, 179.26, 160.94
Phenylpropanoid glycoside	2.45	284.4, 324.4	–	639.35	639.54, 621.35, 487.41, 477.21, 469.35, 459.46, 323.2, 179.13, 161.07
Cycloolivil	2.53	227.5, 279.2	–	375.52	^b^359.38; 341.35; 323.32; 311.36
Isoverbascoside	2.56	286.1, 330.4	–	623.67	623.43, 461.12, 315.06, 297.28, 161.90
Crenatoside	2.75	330.5	623.67	621.63	621.41, 459.37, 178.89, 160.89
Verbascoside	2.81	287.5, 327.4	–	623.72	623.24, 461.06, 315.12, 161.15
Isocrenatoside	3.11	284.2, 327.4	–	621.64	621.41, 459.12, 179.07, 160.89
Leucosceptoside A	3.21	284.4, 323.5	–	637.22	637.21, 501.26, 461.23, 175.16, 161.12
Paulownin	4.73	232.1, 285.3	371.53	–	^b^353.43; 325.46; 323.38; 151.15
Paulownin acetate	5.71	237.4, 286.1	413.48	–	^b^353.23; 335.27; 325.27; 323.25
Sesamin	5.91	235.1, 286.1	355.26	–	^b^337.29; 335.40; 319.26; 307.37
***T. stans*var*angustata*(leaves)**					
Isoverbascoside	2.57	327.4	–	623.59	623.28, 461.17, 315.23, 297.23, 161.77
Crenatoside	2.75	327.5	–	621.57	621.34, 459.62, 178.95, 161.01
Apigenin	4.36	267.4, 331.5	271.37	269.22	268.94, 151.12, 117.08
Methoxyluteolin	4.51	267.5, 345.5	301.43	299.40	299.06, 284.25, 256.03, 150.81, 147.95
***T. stans*var*angustata*(trunk)**					
Olivil	2.23	283.1	–	375.52	^b^359.45; 341.28; 323.17; 311.43
Unidentified	2.35	280.3	–	581.65	581.25, 419.36, 401.17, 233.10, 152.96, 118.87
Isoverbascoside	2.57	330.1	625.68	623.66	623.30, 461.37, 315.0, 297.03, 161.90
Crenatoside	2.75	330.5	–	621.63	621.34, 459.62, 178.95, 161.01
Verbascoside	2.81	287.4, 327.2	625.75	623.66	623.24, 461.0, 315.12, 161.03
Isocrenatoside	3.11	285.3, 327.5	–	621.63	621.46, 459.06, 178.82, 161.01
Phenylpropanoid glycoside	3.23	284.1, 325.4	–	637.64	637.52, 490.93, 475.20, 315.21, 175.22, 161.19
Isomartynoside/Martynoside	3.36	286.5, 327.2	–	651.58	651.13, 475.31, 328.79, 174.92, 160.0
Paulownin	4.73	232.2, 285.1	371.55	–	^b^353.43; 325.46; 323.38; 151.15
Paulownin acetate	5.71	237.5, 286.2	413.47	–	^b^353.23; 335.27; 325.27; 323.25
Sesamin	5.91	235.4, 286.3	355.28	–	^b^337.29; 335.40; 319.26; 307.37
***T. castaneifolia*(leaves)**					
Unidentified	2.35	281.4	–	581.65	581.18, 419.02, 401.4, 232.94, 152.94, 118.91
Isoverbascoside	2.56	285.1, 330.5	625.75	623.66	623.12, 461.12, 315.19, 297.09, 161.0
Verbascoside	2.81	285.3, 327.4	625.68	623.59	623.37, 461.30, 315.12, 161.0
***T. castaneifolia*(trunk)**					
Olivil	2.23	283.5	–	375.52	^b^359.30; 341.39; 323.17; 311.43
Unidentified	2.35	280.3	–	581.71	581.21, 419.12, 401.35, 232.89, 152.97, 118.56
Isoverbascoside	2.57	286.1, 330.3	–	623.66	623.12, 461.12, 315.19, 297.09, 161.0
***T. castaneifolia*(trunk)**					
Crenatoside	2.75	330.5	–	621.63	621.34, 459.62, 178.95, 161.01
Verbascoside	2.81	287.2, 326.5	–	623.66	623.37, 461.30, 315.12, 161.0
Isocrenatoside	3.11	282.3, 326.6	–	621.64	621.46, 459.06, 178.82, 161.01
Isomartynoside/Martynoside	3.36	284.2, 323.5	–	651.55	651.31, 475.51, 329.46, 175.02, 160.91
Paulownin	4.73	232.3, 285.1	371.46	–	^b^353.43; 325.46; 323.38; 151.15
***T. garrocha*(leaves)**					
Isoverbascoside	2.57	326.5	625.68	623.66	623.37, 461.12, 315.0, 297.47, 161.03
Rutin	2.69	255.2, 359.3	611.77	609.61	609.09, 301.11
Verbascoside	2.81	314.6	625.62	623.66	623.31, 461.43, 314.78, 161.0
Phenylpropanoid glycoside	3.23	327.1	639.60	637.83	637.46, 461.23, 443.60, 314.83, 175.17, 160.56
Methoxyluteolin glycoside	3.32	265.3, 331.4	477.48	475.55	^b^477.50, 315.65, 301.36
***T. garrocha*(trunk)**					
Unidentified	2.35	280.5	–	581.65	581.13, 419.17, 401.8, 232.97, 152.99, 118.94
Isoverbascoside	2.56	330.5	625.68	623.66	623.37, 461.12, 315.0, 297.47, 161.03
Verbascoside	2.81	326.4	625.88	623.64	623.31, 461.43, 314.78, 161.0

^a^(−) ions undetected

^b^Characteristic m/z of ions in positive ion mode

There were some phenylethanoid glycosides found in the ethanolic extracts of the *Tecoma* species. These molecules were detected and partially characterized by UPLC-MS. The analysis of the extract obtained from the *T. stans* var. *stans* trunk detected compounds with retention times (Rt) of 2.43 and 3.21 min with molar mass of 638 Da. A full-scan mass spectrum in the negative mode detected deprotonated molecules of m/z 637.21 and 637.22, respectively. Further analysis of the fragment ions by the MS^2^ experiment suggest the presence of feluric acid residue in two compounds.

**Fig. 4:**
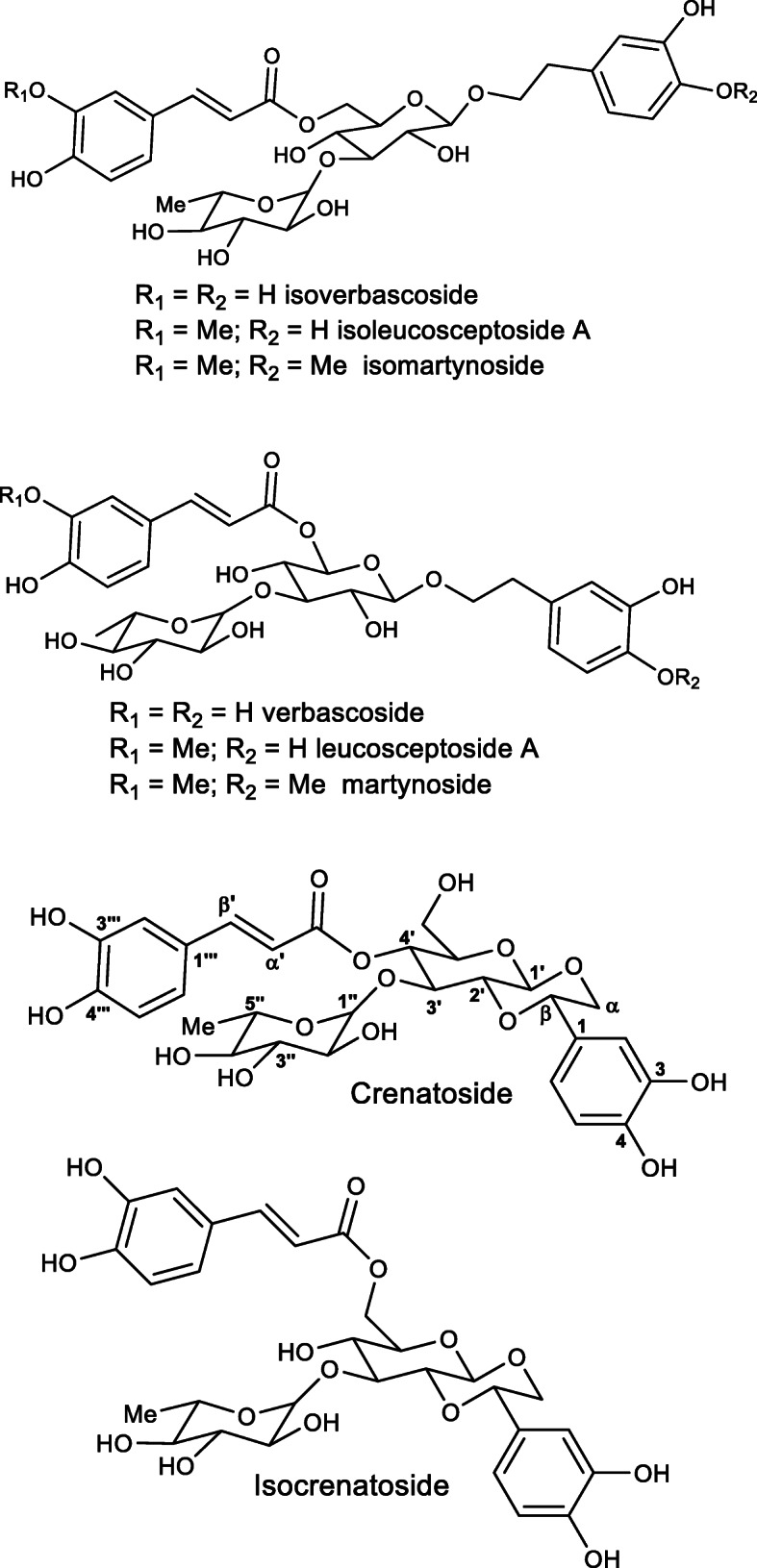
Chemical structures of phenylethanoids glycoside from the leaves and trunk of *Tecoma* species

The compounds with similar chemical structures were also detected in the extracts of *T. stans* var. *angustata* trunks and in the extract of *T. garrocha* leaves (Table 2). There were two phenylethanoids (Rt 2.75 and 3.11 min) showing 622 Da, which were detected in the extracts of *T. stans* var. *stans* trunk, *T. stans* var. *angustata* trunk and leaves, and in the *T. castaneifolia* trunk (Table 2). The ion fragments, obtained by the MS^2^ experiment, suggested that these compounds have similar structures to crenatoside (Fig. 4). A full-scan mass spectra in the trunk extracts of *T. stans* var. *angustata* and *T. castaneifolia* negative mode detected deprotonated molecules of m/z 621.63 and 621.64, respectively. Comparing the data obtained in the MS^2^ experiments and the literature, it is suggested that compounds with Rt 2.75/3.11 have a similar structure to isocrenatoside/crenatoside.

Lignans were also detected in *T. stans* var. *stans*, *T. stans* var. *angustata* and *T. castaneifolia* trunk extracts. The olivil was present in three extracts. The paulownin, sesamin and paulownin acetate were found in extracts of two varieties of *T. stans*. Lastly, cycloolivil was only detected in the extract of *T. stans* var. *stans*.

Other phenolic compounds were identified in the leaves extracts of *T. stans* var. *angustata* and *T. garrocha*. Co-injection analyzes with authentic samples allowed for the identification of two flavones: Apigenin as constituent of *T. stans* var. *angustata* extract and rutin as constituent of *T. garrocha* extract. In addition, two different flavones with 300 Da molar mass and 476 Da were detected in the leaves extracts of *T. stans* var. *angustata* and *T. garrocha*, partially characterized as methoxyluteolin and methoxyluteolin glycoside, respectively.

### Bioguided fractionation and identification of crenatoside isolated from trunk ethanolic extract of *T. stans* var. *stans*

Trunk ethanolic extract of *T. stans* var. *stans* was fractionated by liquid-liquid partition employing sequential extractions of its aqueous methanol solution with immiscible solvents (dichloromethane and ethyl acetate) led to three fractions: dichloromethane fraction (TSSDF, 13.4 g), ethyl acetate fraction (TSSEF, 19.8 g) and aqueous fraction (TSSAF, 12.3 g). TSSEF and TSSAF fractions were activity against ZIKV with EC_50_ values of 149.90 and 78.98 μg/mL, respectively. The ethyl acetate fraction showed moderate antiviral activity (EC_50_ 149.9 μg/mL) and preliminary phytochemical investigation by TLC was observed in the presence of phenolic compounds such as lignans and phenylethanoid glycosides (data not shown). Thus, a fraction of ethyl acetate was chosen to be fractionated.

The ethyl acetate fraction (8.0 g) was further fractionated by silica chromatography allowing isolation of crenatoside (110.7 mg). This phenylethanoid glycoside was identified by ^13^C and ^1^H NMR data that are shown in the Supplementary Material (Fig. 9S to 15S). The spectra of the compounds isolated showed in the sensitivity was higher when chromatograms were acquired in negative-ion mode mass spectra in the Supplementary Material (Fig. 1S to 8S) and it was confirmed using the ^1^H and ^13^C NMR previously reported data for crenatoside [21, 22]. The structure of crenatoside obtained from ethyl acetate fraction is shown in Fig. 4.

### Antiviral assay of *Tecoma* species extract and constituent

The ethanolic extracts of species from genus *Tecoma*, *T. stans* var. *stans*, *T. stans* var. *angustata*, *T. castaneifolia* and *T. garrocha*, were evaluated for in vitro antiviral against the *Zika virus* in Vero cell line, in non-cytotoxic concentrations. In the cytotoxic concentrations (CC_50_) of the extracts, the fractions from trunk ethanol extract of *T. stans* and crenatoside were previously determined at concentrations ranging from 200 to 0.125 μg/mL. The trunk extracts were more cytotoxic than the respective leaves extracts. The extracts from the leaves and the fractions from *T. stans* were not cytotoxic in the highest concentration tested (200.0 μg/mL), while the CC_50_ of the trunk extracts ranged from 159.0 to 0.1954 μg/mL, in Vero cell line. Similarly, the CC_50_ determined in MRC-5 cells showed that the trunks are more cytotoxic than the leaves of the respective *Tecoma* species.

The in vitro anti-*Zika virus* activity by MTT assay was determined as the mean effective concentration (EC_50_), and the trunk extract of *T. castaneifolia* presented EC_50_ of 66.78 μg/mL, while the leaf extract presented EC_50_ of 61.25 μg/mL, with selectivity indexes of 1.53 and 3.27, respectively. The *T. garrocha* specie showed to be less active. This trunk extract gave EC_50_ of 131.0 μg/mL and the leaves extract presented EC_50_ of 149.90 μg/mL with SI less than 1.5. The trunk extract of *T. stans* var. *angustata* and *T. stans* var. *stans* were not active against the *Zika virus*. Nevertheless, the analysis of the leaves extracts from these two specimens indicated activity with EC_50_ below 100.0 μg/mL. Thus, the *T. stans* var. *angustata* extract showed a better antiviral activity with EC_50_ of 53.62 μg/mL followed by *T. stans* var. *stans* extract with a moderate activity of EC_50_ of 98.39 μg/mL and selectivity indexes greater than 3.73 and 2.03, respectively.

The ethyl acetate (TSSEF) and aqueous (TSSAF) fractions of *T. stans* var. *stans* trunk ethanolic extract was active against the *Zika virus* with EC_50_ values of 149.90 and 78.98 μg/mL, and selectivity indexes of 1.33 and 2.53, respectively.

The crenatoside, isolated from the ethyl acetate fraction of the *T. stans* var. *stans* trunk ethanolic extract presented better activity than the origin extract and the ethyl acetate fraction with EC_50_ of 34.78 μM and SI of 4.25. In this assay, ribavirin was used as positive control and it presented an EC_50_ of 386.85 μM and SI of 3.92.

The results including effective concentrations (EC_50_) from each extract, isolated compound and positive control are described in Table 3, as well as the results of cytotoxicity concentrations (CC_50_) for Vero and MRC-5 cell lines.

**Table 3:** Antiviral (EC_50_) and cytotoxic (CC_50_) activity of *Tecoma* species extract and constituents

Extract/compound		MRC-5	Vero	ZIKV	^c^SI
		CC_50_ μg/mL (μM) ^a^	CC_50_ μg/mL (μM) ^a^	EC_**50**_ μg/mL (μM) ^b^	
***Tecoma castaneifolia***	Trunk	131.30 ± 1.39	102.20 ± 2.15	66.78 ± 3.01	1.53
	Leaves	> 200.00	> 200.00	61.25 ± 2.65	> 3.27
***Tecoma garrocha***	Trunk	> 200.00	159.00 ± 1.38	131.00 ± 1.56	1.21
	Leaves	> 200.00	> 200.00	149.90 ± 1.52	> 1.33
***Tecoma stans*var.*angustata***	Trunk	80.25 ± 1.18	0.4750 ± 1.28	> 200.00	–
	Leaves	144.10 ± 1.33	> 200.00	53.62 ± 3.25	> 3.73
***Tecoma stans*var.*stans***	Trunk	0.4258 ± 1.30	0.1934 ± 1.22	> 200.00	–
	Leaves	> 200.00	> 200.00	98.39 ± 1.40	> 2.03
**TSSDF**		NT ^d^	86.94 ± 2.08	> 200.00	–
**TSSEF**		NT ^d^	> 200.00	149.90 ± 3.03	1.33
**TSSAF**		NT ^d^	> 200.00	78.98 ± 1.54	2.53
**Crenatoside**		> 100.00 (> 160.74)	92.06 ± 1.47 (147.98 ± 1.47)	21.64 ± 1.57 (34.78 ± 1.57)	4.25
**Ribavirin**		52.66 ± 1.18 (215.65 ± 1.18)	370.40 ± 1.20 (1516.75 ± 1.20)	94.47 ± 2.70 (386.85 ± 2.70)	3.92

^a^ 50% cytotoxic concentration

^b^ 50% effective concentration of viral replication

^c^ SI (Selectivity index): ratio between extract/compound CC_50_ and EC_50_

^d^ NT: Not tested

The selectivity index (SI) can be a parameter selection to predict promising drugs, it reflects the potency and possible selectivity for future drug development. The SI for active ethanolic extracts ranged from 1.21 to 3.73. The ribavirin used as a positive control showed a SI of 3.92, while the isolated compound crenatoside showed better SI of 4.25 revealing its potent in vitro anti-*Zika virus* activity.

**Fig. 5:**
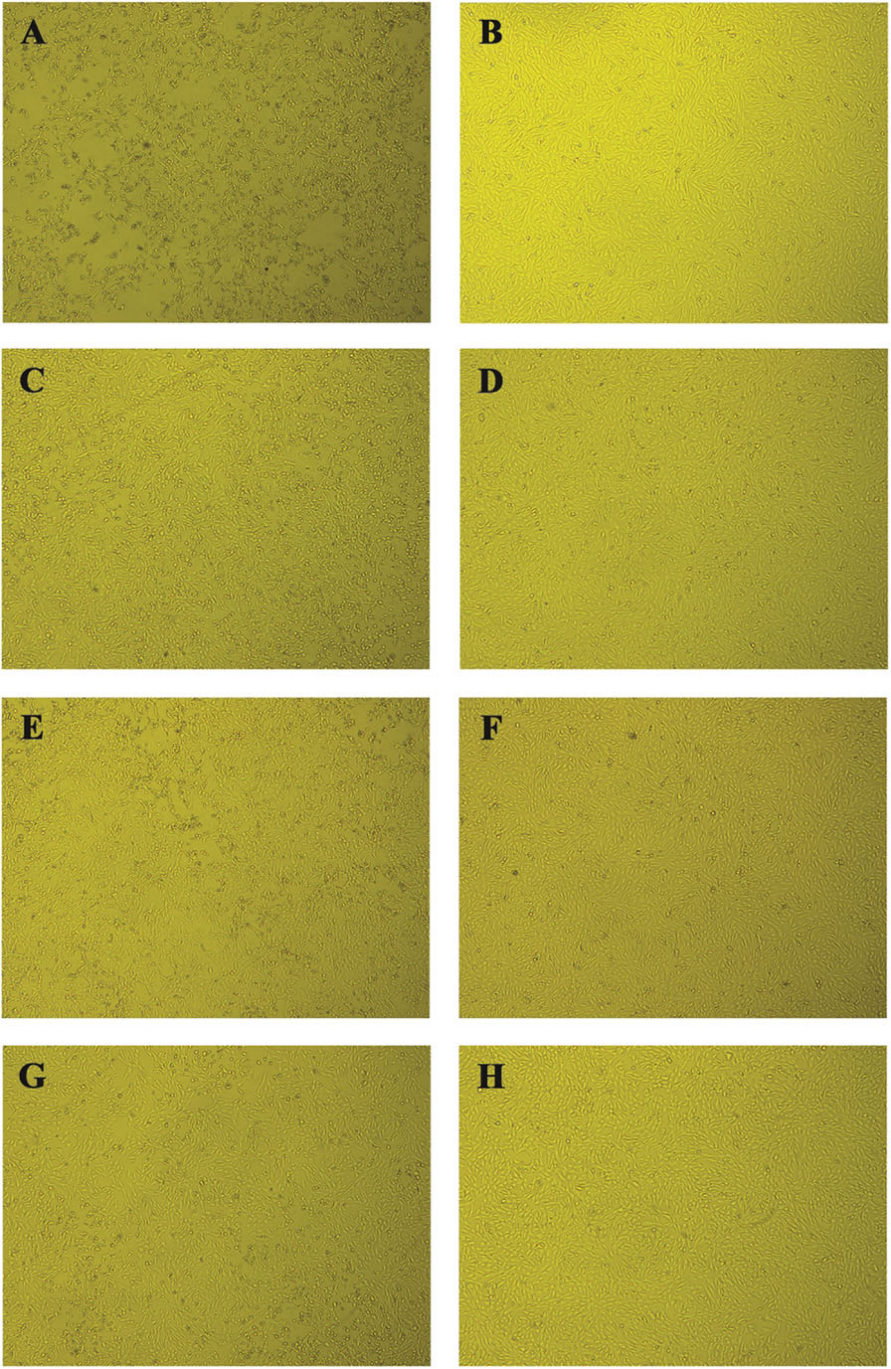
Antiviral effect against *Zika virus* in Vero cells treated with *Tecoma* species leaves extracts. Vero cells were infected with ZIKV, treated with *Tecoma* leaves ethanolic extracts and photographed after 24 h of infection. **a** Infected cells, **b** Uninfected and untreated cells, **c** Cells infected and treated with *T. castaneifolia* leaf (50 μg/mL), **d** Cells uninfected and treated with *T. castaneifolia* leaf (50 μg/mL), **e** Cells infected and treated with *T. garrocha* leaf (200 μg/mL), **f**. Cells uninfected and treated with *T. garrocha* leaf (200 μg/mL), **g** Cells infected and treated with *T. stans* var*. angustata* leaf (50 μg/mL), **h** Cells uninfected and treated with *T. stans* var*. angustata* leaf (50 μg/mL), Magnification, 100x

**Fig. 6:**
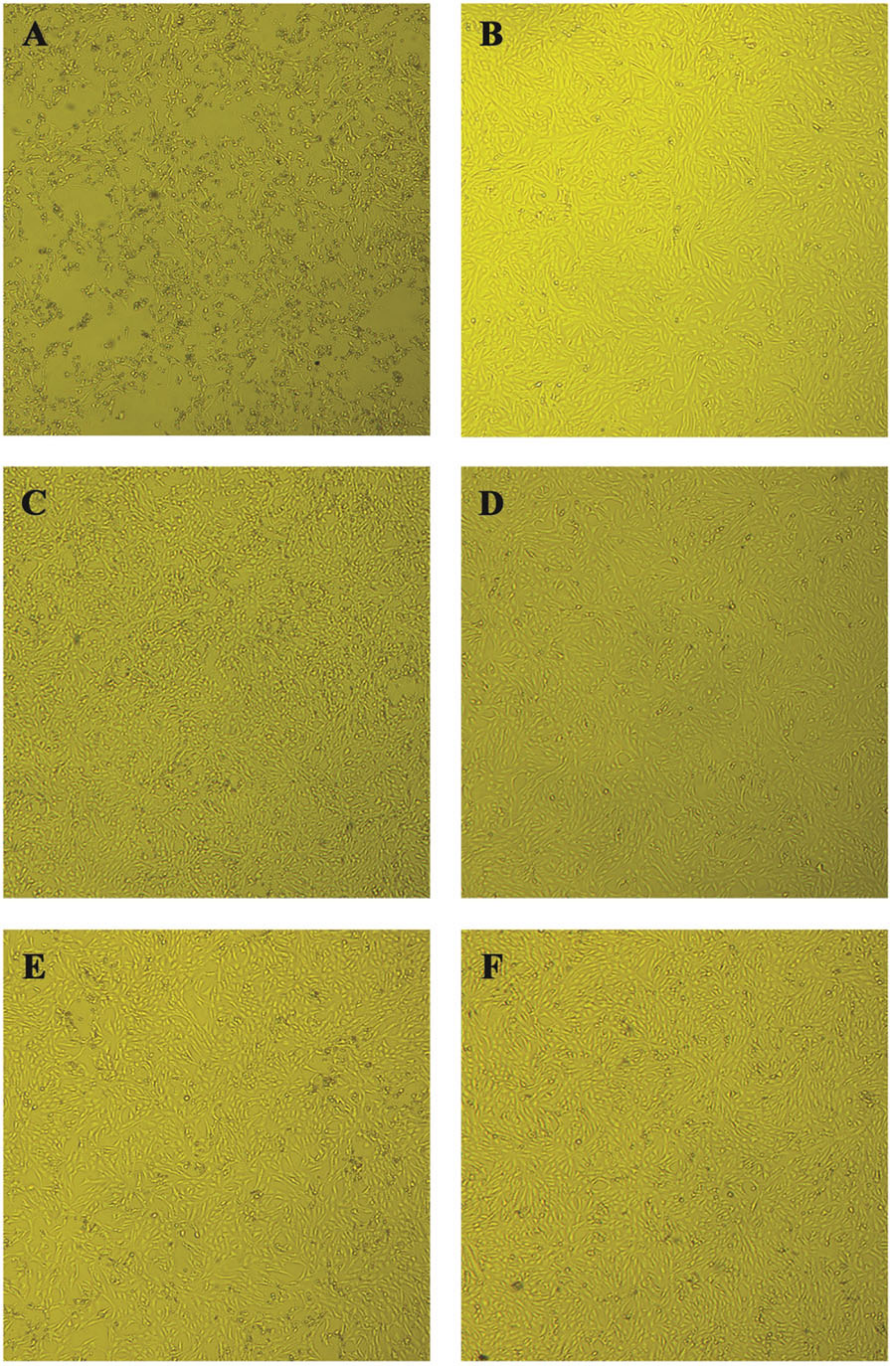
Antiviral effect against *Zika virus* in Vero cells treated with *Tecoma* species trunk extracts. Vero cells were infected with ZIKV, treated with *Tecoma* trunk ethanolic extracts and photographed after 24 h of infection. **a** Infected cells, **b** Uninfected and untreated cells, **c** Cells infected and treated with *T. castaneifolia* trunk (50 μg/mL), **d** Cells uninfected and treated with *T. castaneifolia* trunk (50 μg/mL), **e** Cells infected and treated with *T. garrocha* trunk (200 μg/mL), **f** Cells uninfected and treated with *T. garrocha* trunk (200 μg/mL). Magnification, 100x

**Fig. 7:**
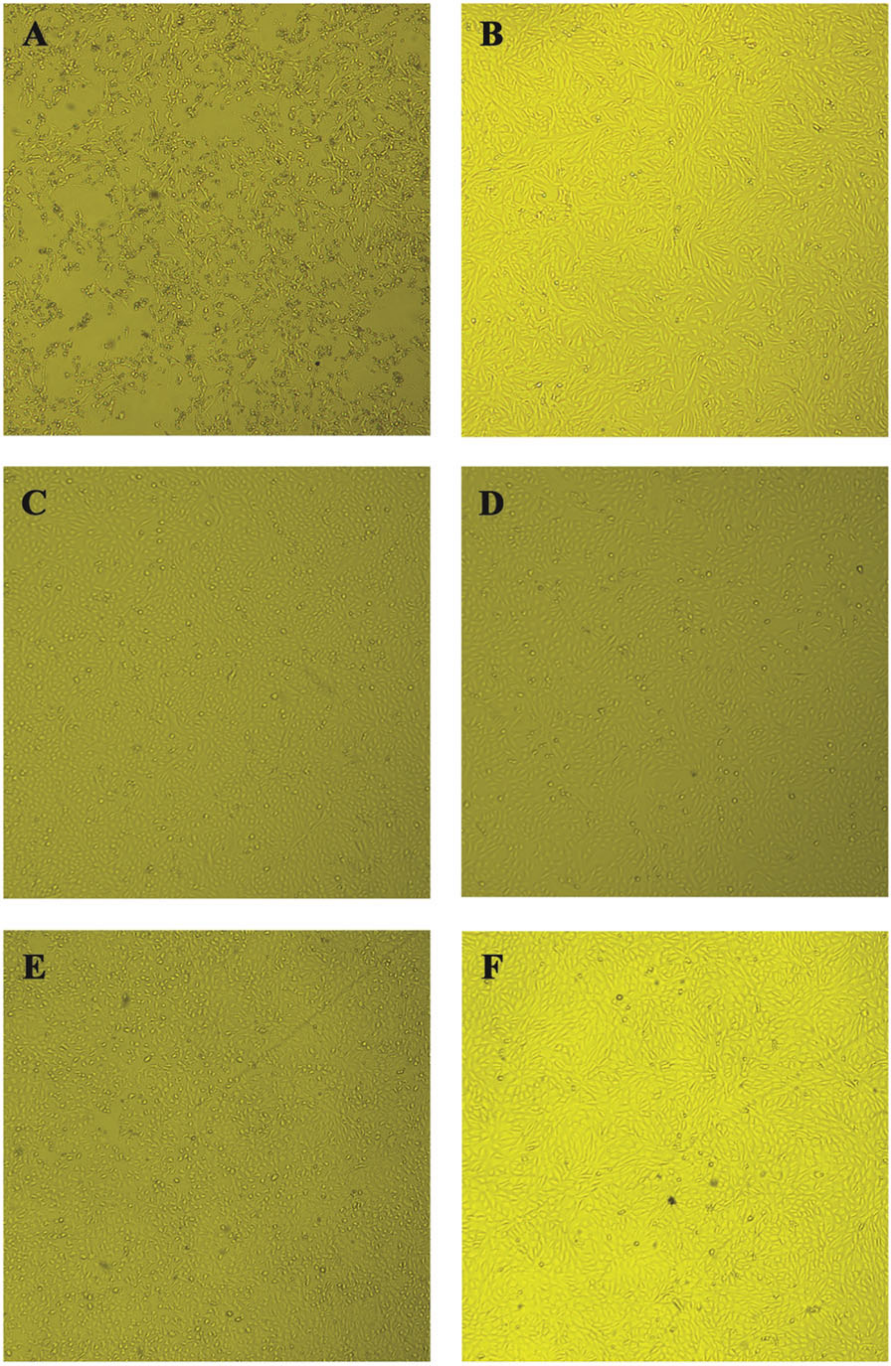
Antiviral effect against *Zika virus* in Vero cells treated with Crenatoside and the positive control Ribavirine. Vero cells were infected with ZIKV, treated with the isolated compound, crenatoside, and the positive control, ribavirine, then photographed after 24 h of infection. **a** Infected cells, **b** Uninfected and untreated cells, **c** Cells infected and treated with crenatoside (25 μg/mL), **d** Cells uninfected and treated with crenatoside (25 μg/mL), **e** Cells infected and treated with ribavirine (100 μg/mL), **f** Cells uninfected and treated with ribavirine (100 μg/mL). Magnification, 100x

In order to confirm the anti-*Zika virus* activity observed in the MTT assay, extracts and isolated crenatoside compound were subjected to an in vitro cytopathic effect inhibition assay. In these experiments, challenges were performed where the extracts and compounds were tested in fixed concentrations. After 24 h, the presence or absence of viral cytopathic effect was observed under an optical microscope (Figs. 5, 6 and 7). In Figs. 5, 6 and 7, it was possible to observe that every extract and isolated compound inhibit the viral cytopathic effect. They showed antiviral activity with monolayer cell protection greater than 80% for all tested substances when compared to the viral control.

## Discussion

The phytochemical investigation of ethanolic extracts in the *Tecoma* species showed the presence of phenolic compounds such as lignans, phenylethanoids glycosides and flavonoids. The trunks ethanolic extracts from all studied species were more cytotoxic when compared to the extracts of the respective leaves, while the highlights were the trunk extracts in two varieties of *T. stans*. The analyzes by UPLC-DAD-MS showed the presence of lignans in these two extracts. In addition, it has been described in the literature that, in general, the antiviral effects of lignans are not strong [23]. The presence of lignans in the extracts of the two varieties of *T. stans* studied could justify the greater cytotoxicity observed in these extracts when compared to the extracts of the other *Tecoma* species tested and the non-activity against the *Zika virus*.

Phenylethanoid glycosides are present in all extracts of the *Tecoma* species from this present study. Literature reports only low to moderate cytotoxicity for this class of compounds [24]. Leaves extracts of the *Tecoma* species presented only low cytotoxicity despite high levels of phenylethanoids glycosides. The anti-*Zika virus* activity was observed for all *Tecoma* leaves extracts and almost all trunk extracts, except for the *T. stans* varieties.

In the literature, there are reports of the antiviral activity of the phenylethanoids glycosides verbascoside and isoverbascoside that were characterized in all *Tecoma* species. The verbascoside and isoverbascoside showed a potent in vitro activity against *Human Immunodeficiency virus type 1* (HIV-1) with EC_50_ of 7.8 and 13.7 μM, respectively [25]. Furthermore, verbascoside was evaluated against *Herpes Simplex virus* exhibiting EC_50_ values of 58 μg/mL (92.95 μM) to HSV-1 and 8.9 μg/mL to HSV-2 (14.26 μM) [26]. Therefore, the data suggest that the antiviral activity against the *Zika virus* may be related to the presence of the characterized phenylethanoids glycosides in *Tecoma* species.

Flavonoids such as rutin, apigenin and methoxyluteolin were detected in leaf extracts of *T. garrocha* and *T. stans* var. *angustata*. The protective effect during plant tissue injury is widespread by antioxidant activity of these compounds [27]. Rutin was identified in leaves extract from *T. garrocha* among other flavonoids. It has been distinguished by various pharmacological activities [28]. According to studies by Afanas’Ev et al.*,* rutin and quercetin, showed antioxidant activity. Therapeutic action of these two flavonoids in pathologies involving free radicals are non-toxic, especially in rutin [29]. There are no studies in the literature demonstrating the antiviral activity of rutin. However, there are reports that associate the antioxidant activity of substances with antiviral activity [20, 30]. Thus, the presence of rutin in the species *T. garrocha* may be responsible for the contribution to the activity of the anti-*Zika virus* observed in this species.

In addition, the rich fraction of *Cynodon dactylon* containing luteolin and apigenin as the major phytochemicals exhibited a potent viral inhibitory activity (about 98%) at a concentration of 50 μg/mL against the *Chikungunya virus* [31]. From this, it is possible to suggest that the presence of apigenin and methoxyluteolin in the species *T. stans* var. *angustata* may contribute to the antiviral activity against the *Zika virus* observed.

The biofractionation of *T. stans* var. *stans* trunk extract led to the isolation of crenatoside that was obtained from the ethyl acetate fraction (TSSEF). It has been identified employing usual spectrometric and spectroscopy techniques followed by comparison with the literature [21, 22], the ^13^C and ^1^H NMR and DEPT spectra data and monography are shown in the Supplementary Material (Fig. 9S to 15S).

Crenatoside is a phenylethanoid glycoside containing glycosidic linkage, as well as an ether linkage between a glucose moiety and a 3,4-dihydroxyphenylethanol moiety [32]. Phenylethanoid glycoside moiety was considered a chemical marker of the *Orobanche* species [33], usually called orobanchoside and oraposide. According to Nishibe et al.*,* [34], these two named structures are the same molecule. In the Bignoniaceae family, its occurrence was reported in *Incarvillea compacta* [35]. This was the first one reported of its isolation in *Tecoma* species.

The zika fever emerged as a threat in the western hemisphere in 2015 and the peak of the *Zika virus* infection occurred in 2016, and over the following years the cases decreased substantially in the Americas region [36, 37]. In mid-2019, the WHO data revealed that 87 countries and territories worldwide recorded autochthonous *Zika virus* transmission by mosquito-borne [1]. However, there is a probable risk of Zika infection to spread to more countries, as well as chances for the potential re-emergence of the virus in all places where ZIKV transmission has been reported previously [37].

Therefore, from the aforementioned, the Zika fever remains a global health threat and it has the potential to re-emerge as an epidemic, furthermore there are no effective vaccine and/or antiviral drugs to prevent or treat Zika infection [37]. In this context, this study proposed to evaluate the anti-*Zika virus* activity of ethanol extracts from *Tecoma* species and promising isolated compound.

The evaluation of antiviral activity against *Zika virus* by *Tecoma* species shows that leaves extracts were more active than trunk extracts (Table 3). Cos et al. [18] and Ocazionez et al. [19] consider that antiviral activity is selective and relevant with the standard criteria adopted below: CC_50_ ≥ 100.0 μg/mL, the EC_50_ ≤ 50.0 μg/mL and the selectivity index ≥2.0 [18, 19]. Therefore, the leaves extract of *T. castaneifolia*, *T. stans* var. *angustata* and *T. stans* var. *stans* presented a better selectivity index (SI >  2.0). More in depth studies with these species might be really promising in order to further investigate antivirus activity.

This paper describes for the first time, the anti-*Zika virus* activity of these *Tecoma* species. However, in relation to the Tecomeae tribe (Bignoniaceae), there are few reports in the literature about the antiviral activity of the species in the *Tabebuia* and *Tecoma* genera. In the El-Mekkawy et al. [38] study, the methanol extract from the aerial parts of *Tabebuia pentaphylla* was active against HIV-1 at a concentration of 100.0 μg/mL, and presented a weak inhibition (< 50.0%) of the activity of the enzyme reverse transcriptase ribonuclease H. While the methanol extracts of *Tabebuia pentaphylla* and *Tecoma grandis* exhibited moderately inhibition (65.90 and 57.60%, respectively) of the HIV-1 protease.

Other Bignoniaceae family species with antivirus activity have been previously reported including in vitro anti-*Dengue virus* type 2 activity, a flavivirus that has a genetic and serological relationship with the *Zika virus*, of *Fridericia* sp. [5; 8], *Distictella elongate* [39], *Xylofragma myrianthum* [40]. *Markhamia lutea* extract presented in vitro activity against Respiratory syncytial virus [6].

According to this study, the phenylethanoid crenatoside antiviral activity shows that it is a promising substance with in vitro anti-*Zika virus* activity, that is, it presents selective and relevant activity when compared to standard criteria [18, 19]. Although its cytotoxicity (CC_50_) is slightly less than 100 μg/mL in Vero cell line, the crenatoside was not cytotoxic in MRC-5 cells at the highest concentration tested, hence according to the recommendations described by Cos et al. [18]. Thus, crenatoside is a promising substance with antiviral activity.

Comparing the crenatoside antiviral activity with the ethanolic extracts of *Tecoma* species, it was at least 2.5 times more active than the crude extracts, in relation to the origin ethyl acetate fraction, and crenatoside was 4.3 times more active. Furthermore, when compared with the positive control, ribavirin, it was 4.4 times more active against ZIKV. Until today, there is no specific treatment for the Zika fever, therefore, ribavirin was chosen as a positive control because it is an antiviral approved drug used to treat hepatitis C. Like the *Zika virus*, the *Hepatitis C virus* is a member of the Flaviviridae family, and in vitro and in vivo studies have shown the ribavirin potential to inhibit the *Zika virus*.

There were some crenatoside biological activities previously reported such as: antioxidant activity, platelet aggregation, *Respiratory syncytial* inhibition; antihypertensive; analgesic [41], inhibition of aldose reductase; antitremor L-DOPA [42] among others. The only crenatoside antiviral reported activity was against the *Influenza virus* type A [43], which presented an inhibitory effect of 89.81 μg/mL (144.36 μM).

The crenatoside is four times more active against the *Zika virus* than the *Influenza virus* type A, although the *Zika virus* and the *Influenza virus* are viruses enveloped with RNA genetic material [44, 45]. Morphology disparities can explain the different results for these viruses, for example, ZIKV is a flavivirus that contains a single-stranded positive-sense [44], while the *Influenza virus* presents a single-stranded negative-sense and belongs to the Ortomyxoviridae family [45].

The results obtained in the in vitro cytopathic effect inhibition assay demonstrated that the cell monolayer infected by the *Zika virus* allows viral multiplication inside the cells leading to the cell monolayer destruction that was observed in the images (item A - Figs. 5, 6 and 7). These can be seen by the destruction and morphological alterations of the cells such as rounding of cells, formation of lumps and changes in cell refringence, especially when compared to the uninfected and untreated cell monolayer (item B - Figs. 5, 6 and 7).

While treating the ZIKV-infected cell monolayer with extracts and / or the substance alone, it is possible to protect the cells from a virus infection, and thus prevent cell death once the cells remain attached (such as a monolayer) and no morphocellular deformities are observed. Therefore, they indicate that the treatment performed after infection is effective in inhibiting the virus multiplication cycle, confirming the antiviral activity observed in the MTT colorimetric assay.

This shows crenatoside is an interesting promisor drug with selective and relevant in vitro anti-*Zika virus* activity. Moreover, the complex structure of the crenatoside allows for many chemical modifications. Thus, future studies of this molecule might provide relevant data to produce antivirus molecules.

## Conclusions

Our results reveal that *Tecoma* species showed in vitro antiviral activity against the *Zika virus* and crenatoside. The first time it was isolated from the *Tecoma stans* var. *stans*, it exhibited a potent and relevant anti-*Zika virus*, being more active than ribavirin (positive control). This data suggests that crenatoside is a promisor compound against the *Zika virus* and future studies of this molecule might provide relevant data to produce antivirus molecules. Furthermore, the bioactivity of crenatoside should be investigated against other viruses.

The phytochemical investigation of *Tecoma* species allowed the detection of phenylethanoids glycosides such as verbascoside, isoverbascoside and crenatoside in the trunk and leaves, furthermore, some lignans and flavonoids were detected. The biomonitored studies with *Tecoma* species are being carried out by our research group to isolate other bioactive compounds with promising antiviral activity, such as crenatoside.

## Supplementary information

**Additional file 1: Fig.1S.** Mass spectrum in negative mode of *Tecoma castaneifolia* trunk. **Figure** **2S.** Mass spectrum in negative mode of *Tecoma castaneifolia* leaves. **Figure** **3S.** Mass spectrum in negative mode of *Tecoma garrocha* trunk. **Figure** **4S.** Mass spectrum in negative mode of *Tecoma garrocha* leaves. **Figure** **5S.** Mass spectrum in negative mode of *Tecoma stans* var. *angustata* trunk. **Figure** **6S.** Mass spectrum in negative mode of *Tecoma stans* var. *angustata* leaves. **Figure** **7S.** Mass spectrum in negative mode of *Tecoma stans* var. *stans* trunk. **Figure 8S.** Mass spectrum in negative mode of *Tecoma stans* var. *stans* leaves. **Figure 9S.** 1H-NMR spectrum of crenatoside (400 MHz, DMSO-d6 e MeOD. **Figure 10S.** 1H-NMR spectrum expansion 7.7 to 6.3 ppm of crenatoside (400 MHz, DMSO-d6 e MeOD, δ). **Figure 11S.** 1H-NMR spectrum expansion 5.2 to 4.5 ppm of crenatoside (400 MHz, DMSO-d6 e MeOD, δ). **Figure 12S.** 1H-NMR spectrum expansion 4.2 to 3.2 ppm of crenatoside (400 MHz, DMSO-d6 e MeOD, δ). **Figure 13S.** 1H-NMR spectrum expansion 1.3 to 0 ppm of crenatoside (400 MHz, DMSO-d6 e MeOD, δ). **Figure 14S.** 13C-NMR spectrum of crenatoside (100 MHz, DMSO-d6 e MeOD, δ). **Figure 15S.** 13C-NMR spectrum of crenatoside (DEPT-135, 100 MHz, DMSO-d6 e MeOD, δ).

## Data Availability

Not applicable;

## Abbreviations

ATCC: American Type Culture Collection
CC_50_: 50% of Citotoxicity Concentration
CH_2_Cl_2_: Dichloromethane
CPE: cytopathic effect
DAD: Diode Array Detector
DMEM: Dulbecco Modified Eagle Medium
DMSO: dimethyl sulfoxide
DMSO-*d6*: Deuterated dimethyl sulfoxide
ESI: Electrospray Ionization
EtOAc: ethyl acetate
EC_50_: 50% of Effective Concentration
FBS: Fetal Bovine Serum
HIV: *Human Immunodeficiency virus*
HSV: *Herpes simplex virus*
LD: Liquid Chromatography
MeOD: Deuterated methanol
MeOH: Methanol
MOI: Multiplicity Of Infecction
MS: Mass Spectrometry
MTT: 3-(4,5-Diamethylthiazol-2-yl)-2,5-Diphenyltetrazolium Bromide
NMR: Nuclear Magnetic Resonance
NT: Not tested
SI: selectivity index
TCID_50_: 50% tissue culture infective dose
TLC: Thin Layer Chromatography
TSSAF: *Tecoma stans* var. *stans* trunk aqueous fraction
TSSDF: *Tecoma stans* var. *stans* trunk dichloromethane fraction
TSSEF: *Tecoma stans* var. *stans* trunk ethyl acetate fraction
UPLC: Ultra Performance Liquid Chromatography
UV: Ultraviolet
WHO: World Health Organization
ZIKV: *Zika virus*
