# Supplementary material for: Anti-Zika virus activity and chemical characterization by ultra-high performance liquid chromatography (UPLC-DAD-UV-MS) of ethanol extracts in Tecoma species
Source: BMC Complement Med Ther. 2020 Aug 7;20:246. doi: 10.1186/s12906-020-03040-0 (PMC7412813; doi:10.1186/s12906-020-03040-0)
Supplement: Supplementary file 1 — Additional file 1: Fig.1S. Mass spectrum in negative mode of Tecoma castaneifolia trunk. Figure 2S. Mass spectrum in negative mode of Tecoma castaneifolia leaves. Figure 3S. Mass spectrum in negative mode of Tecoma garrocha trunk. Figure 4S. Mass spectrum in negative mode of Tecoma garrocha leaves. Figure 5S. Mass spectrum in negative mode of Tecoma stans var. angustata trunk. Figure 6S. Mass spectrum in negative mode of Tecoma stans var. angustata leaves. Figure 7S. Mass spectrum in negative mode of Tecoma stans var. stans trunk. Figure 8S. Mass spectrum in negative mode of Tecoma stans var. stans leaves. Figure 9S. 1H-NMR spectrum of crenatoside (400 MHz, DMSO-d6 e MeOD. Figure 10S. 1H-NMR spectrum expansion 7.7 to 6.3 ppm of crenatoside (400 MHz, DMSO-d6 e MeOD, δ). Figure 11S. 1H-NMR spectrum expansion 5.2 to 4.5 ppm of crenatoside (400 MHz, DMSO-d6 e MeOD, δ). Figure 12S. 1H-NMR spectrum expansion 4.2 to 3.2 ppm of crenatoside (400 MHz, DMSO-d6 e MeOD, δ). Figure 13S. 1H-NMR spectrum expansion 1.3 to 0 ppm of crenatoside (400 MHz, DMSO-d6 e MeOD, δ). Figure 14S. 13C-NMR spectrum of crenatoside (100 MHz, DMSO-d6 e MeOD, δ). Figure 15S. 13C-NMR spectrum of crenatoside (DEPT-135, 100 MHz, DMSO-d6 e MeOD, δ). [file 12906_2020_3040_MOESM1_ESM.pdf]

Supplementary Material

Title: Anti-Zika virus activity and chemical characterization by Ultra-High-Performance Liquid Chromatography (UPLC-DAD-UV-MS) of ethanol extracts in *Tecoma* species

Adriana Cotta Cardoso Reis:
Pharmacy Department, School of Pharmacy, Federal University of Ouro Preto,
Campus Morro do Cruzeiro, Ouro Preto, Minas Gerais, Brazil.
adrianacotta.r@gmail.com

Breno Mello Silva:
Department of Biological Sciences, ICEB, Federal University of Ouro Preto
Campus Morro do Cruzeiro, Ouro Preto, Minas Gerais, Brazil.
breno@ufop.edu.br

Hélia Maria Marques de Moura:
Pharmacy Department, School of Pharmacy, Federal University of Ouro Preto,
helialenoir@hotmail.com

Guilherme Rocha Pereira:
Department of Physics and Chemistry, Institute of Exact Sciences and IT (ICEI)
Catholic Pontifical University of Minas Gerais, PUC Minas, Belo Horizonte, Minas Gerais, Brazil.
guilhermepereira2000@yahoo.com

Corresponding author:
Geraldo Célio Brandão:
Pharmacy Department, School of Pharmacy, Federal University of Ouro Preto,
Campus Morro do Cruzeiro, 35400-000, Ouro Preto, Minas Gerais, Brazil. Phone: +55-31-3551-1088; Fax: +55-31-3551-1069.

celiobrandao@ufop.edu.br or celiobrandao@yahoo.com

Mass spectrum of *Tecoma* species

Figure 1S – Mass spectrum in negative mode of *Tecoma castaneifolia* trunk

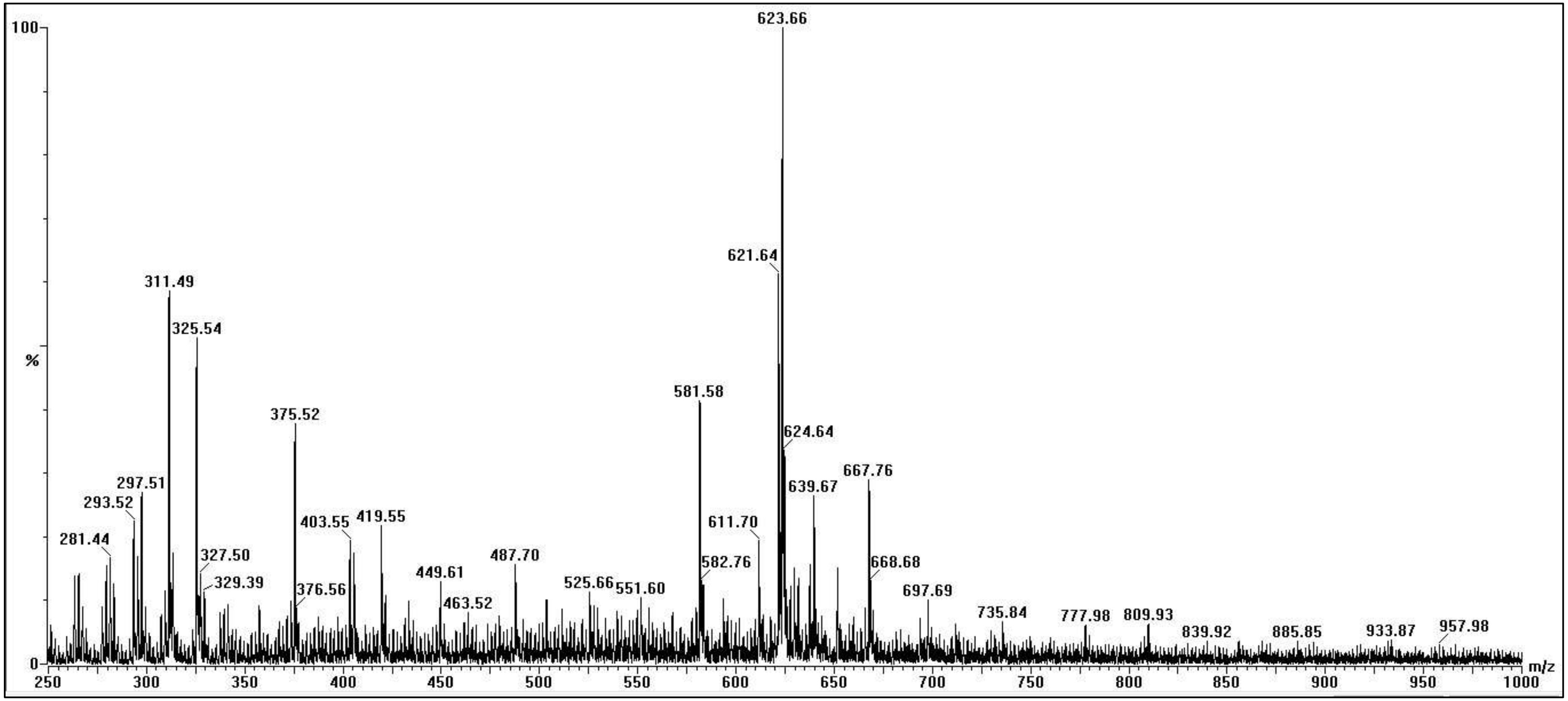

Figure 2S – Mass spectrum in negative mode of *Tecoma castaneifolia* leaves

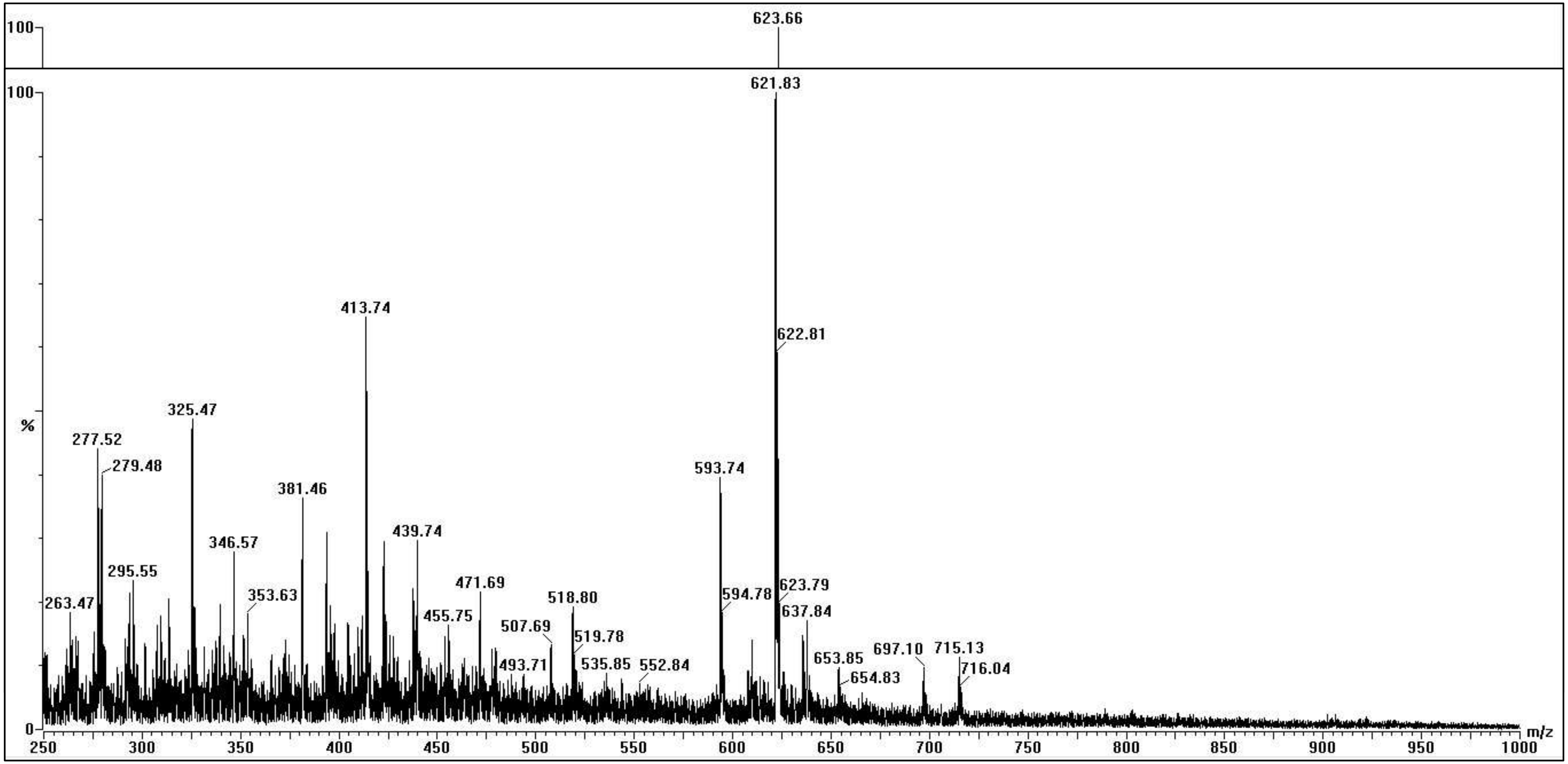

Figure 3S – Mass spectrum in negative mode of *Tecoma garrocha* trunk

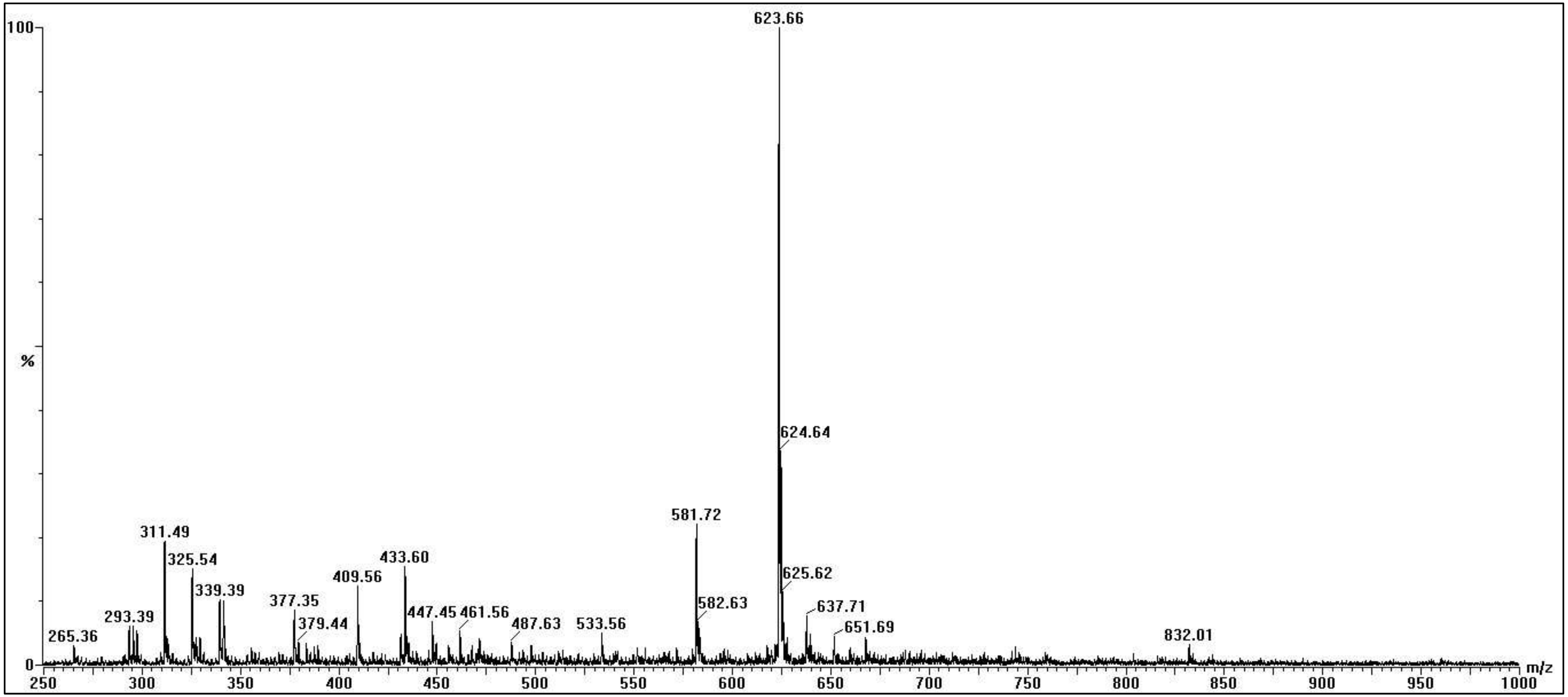

Figure 4S – Mass spectrum in negative mode of *Tecoma garrocha* leaves

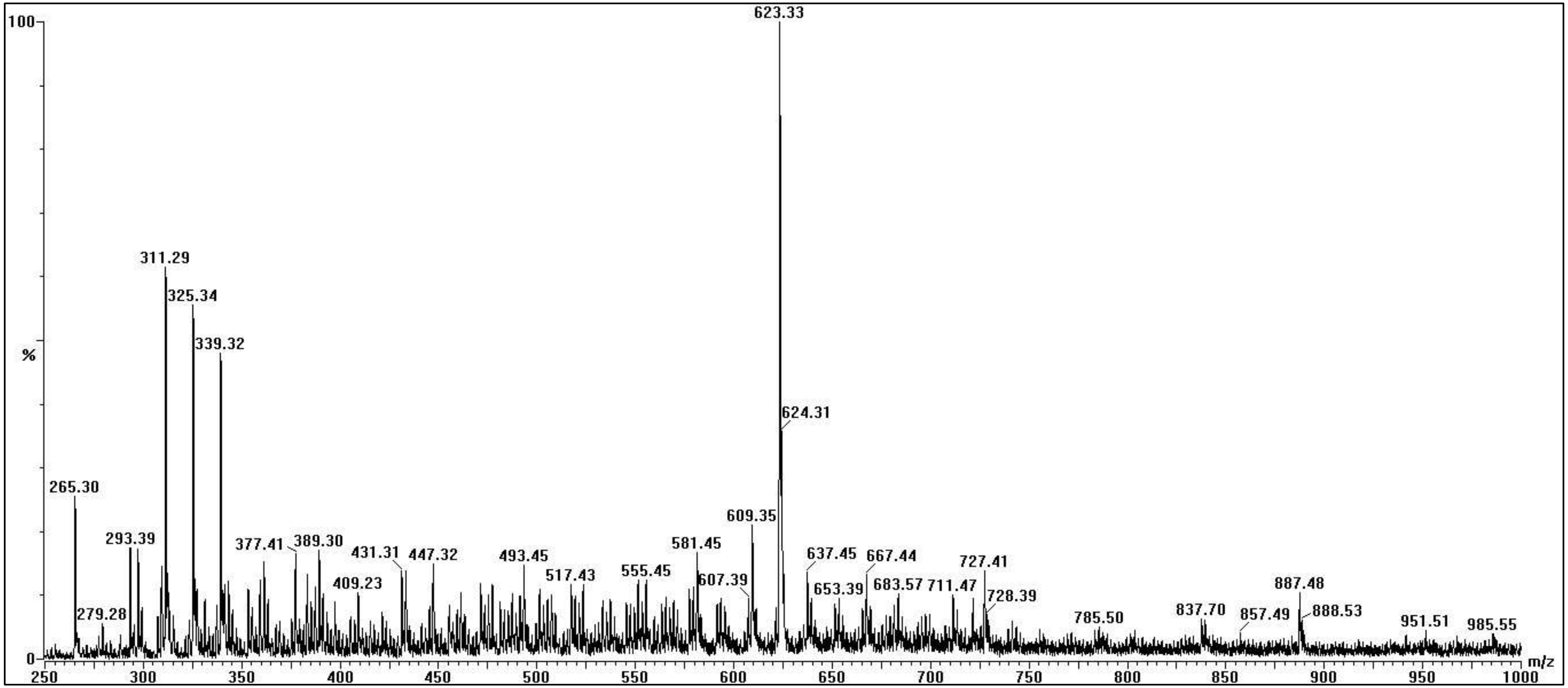

Figure 5S – Mass spectrum in negative mode of *Tecoma stans* var. *angustata* trunk

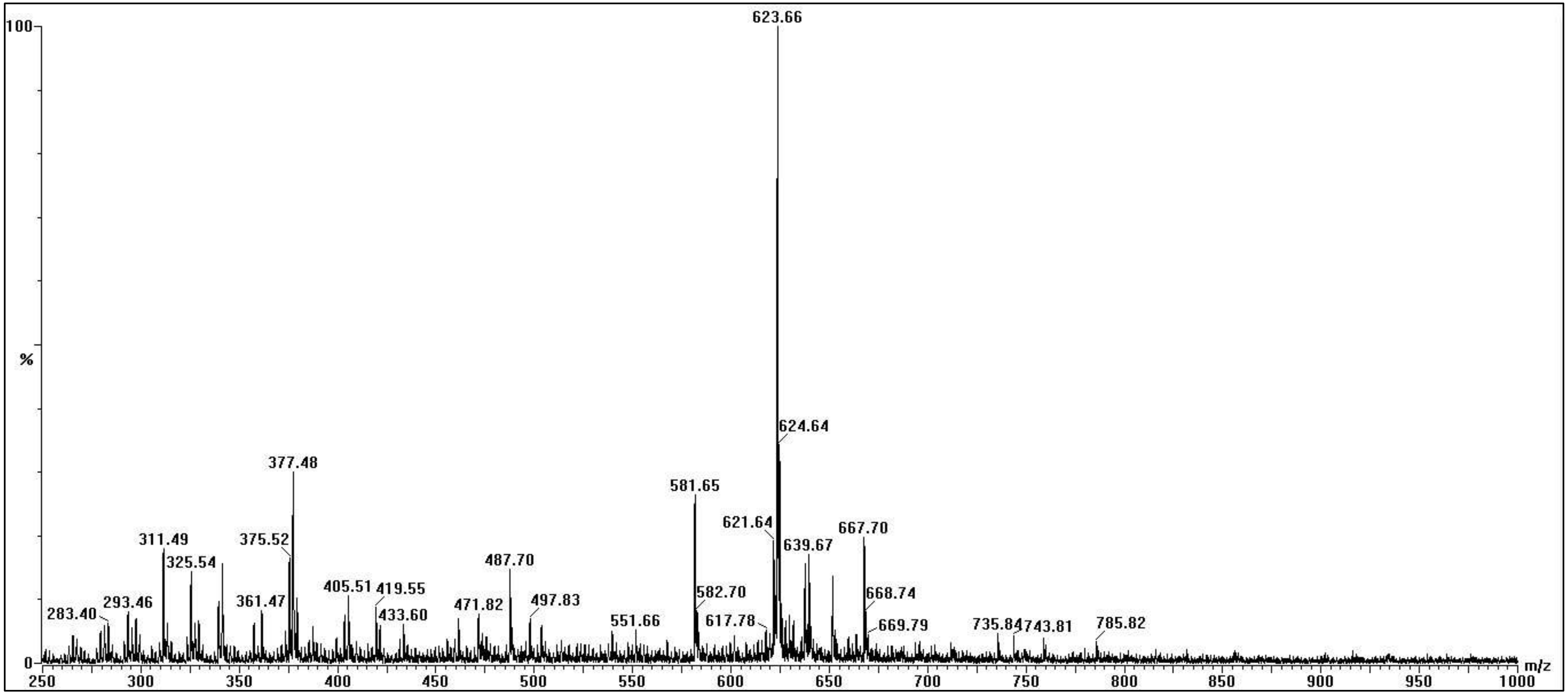

Figure 6S – Mass spectrum in negative mode of *Tecoma stans* var. *angustata* leaves

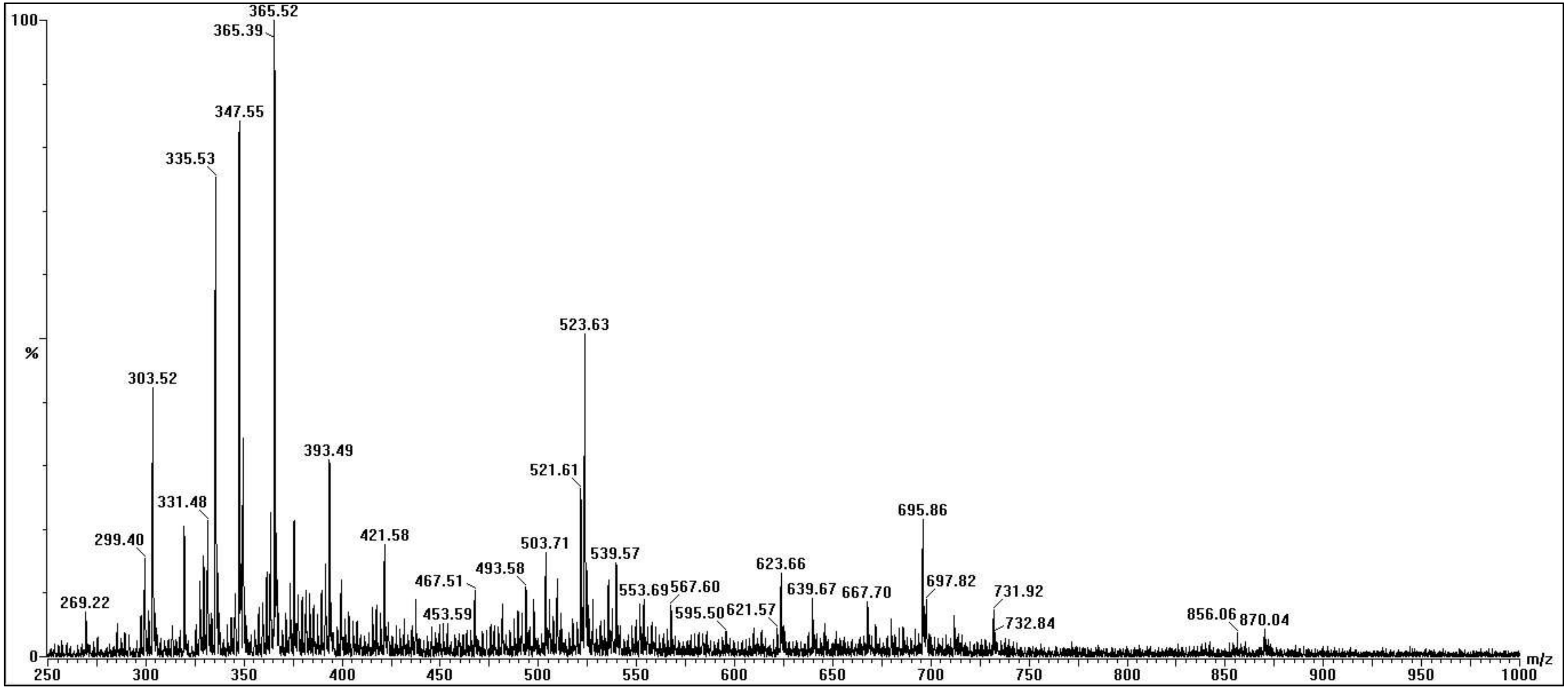

Figure 7S – Mass spectrum in negative mode of *Tecoma stans* var. *stans* trunk

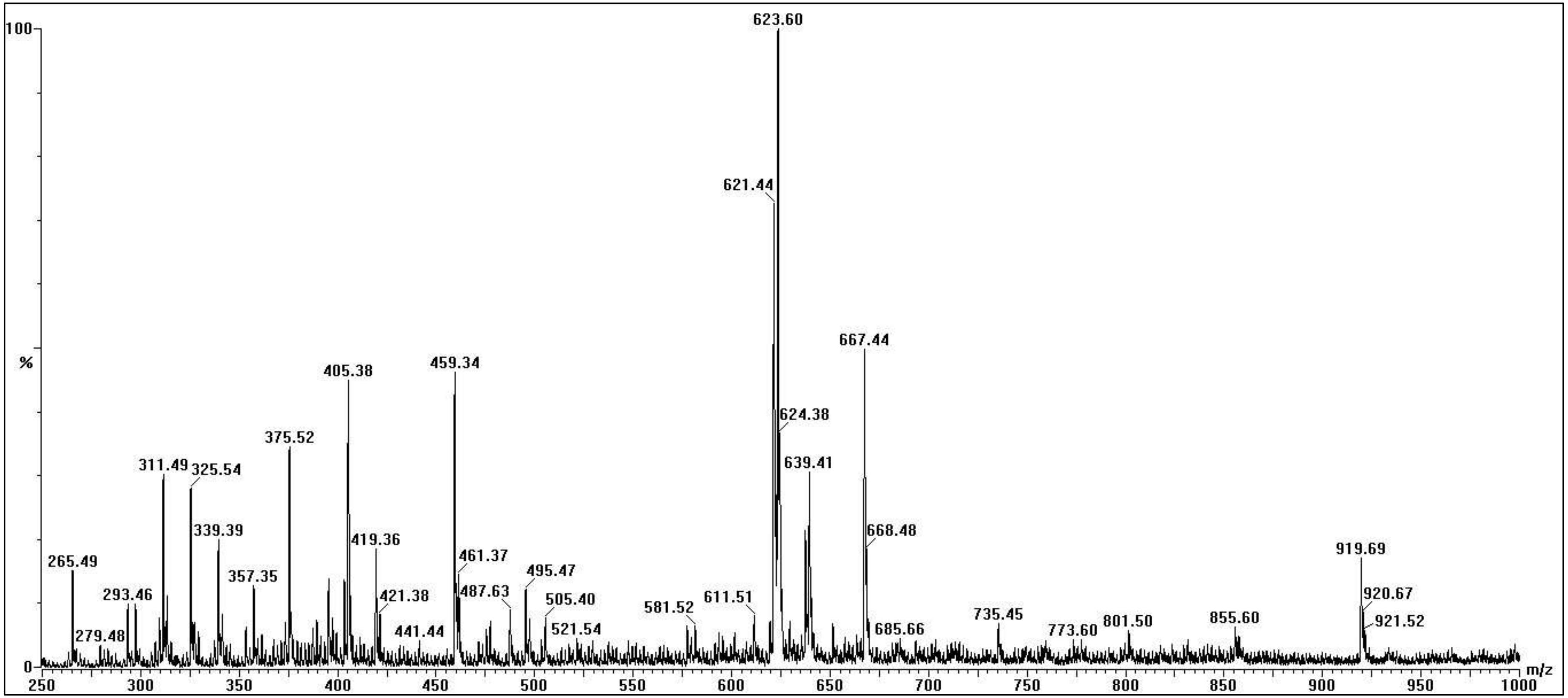

Figure 8S – Mass spectrum in negative mode of *Tecoma stans* var. *stans* leaves

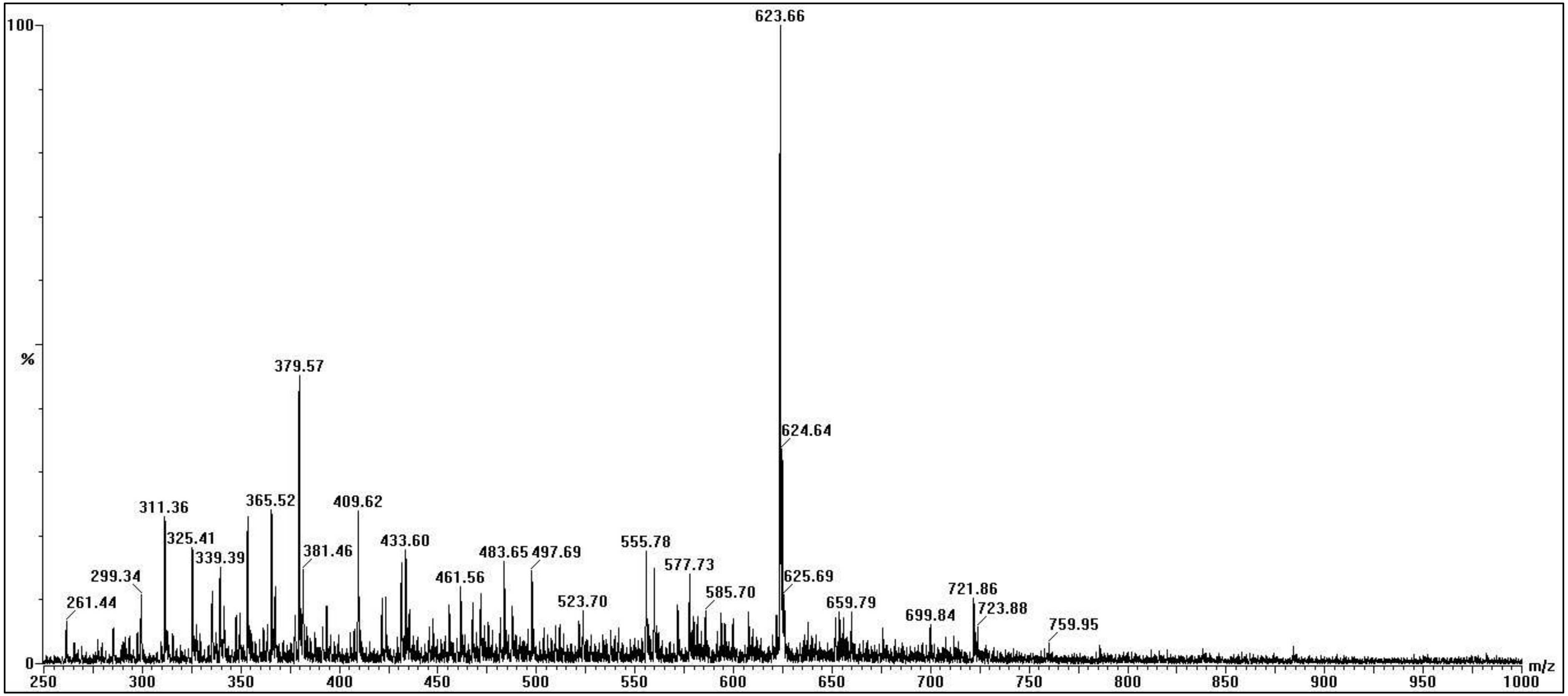

NMR Data for Isolated Compound – Crenatoside

Spectroscopic Data for Crenatoside

Crenatoside: White solid (MeOH); m.p. Lit. 201-202 °C [21]; UV (MeOH) λ_{max} 330.5 nm; ^{1}H NMR (DMSO-*d6* and MeOD, 400 MHz): δ 6.82 (1H, d, *J* = 1.8 Hz, H-2), 6.73 (1H, d, *J* = 8.1 Hz, H-5), 6.69 (1H, dd, *J* = 1.8/8.2 Hz, H-6), 4.60 (1H, dd, *J* = 2.6/10.5, H-β), 3.99 (2H, dd, *J* = 2.9/12.0 Hz, H-α eq), 3.63 (2H, m, H-α ax), 4.55 (1H, d, *J* = 7.8 Hz, H-1'), 3.47 (1H, m, H-2'), 4.13 (1H, t, *J* = 9.4 Hz, H-3'), 5.08 (1H, t, *J* = 9.6 Hz, H-4'), 3.75 (1H, sl, H-5'), 3.65 e 3.55 (1H, m, H-6'), 5.15 (1H, d, *J* = 1.4 Hz, H-1''), 3.77 (1H, sl, H-2''), 3.49 (1H, m, H-3''), 3.25 (1H, t, *J* = 9.5 Hz, H-4''), 3.57 (1H, m, H-5''), 1.11 (1H, d, *J* = 6.2 Hz, H-6''), 7.06 (1H, d, *J* = 2.0 Hz, H-2'''), 6.78 (1H, d, *J* = 8.2 Hz, H-5'''), 6.97 (1H, dd, *J* = 2.0/8.2 Hz, H-4'''), 7.60 (1H, d, *J* = 16.0 Hz, H-β'), 6.28 (1H, d, *J* = 15.6 Hz, H-α'); ^{13}C NMR (DMSO-*d6* and MeOD, 100 MHz): δ 129.8 (C-1), 114.5 (C-2), 146.3 (C-3), 146.3 (C-4), 116.2 (C-5), 118.8 (C-6), 72.7 (C-α), 78.2 (C-β), 98.9 (C-1'), 81.9 (C-2'), 77.0 (C-3'), 70.0 (C-4'), 77.7 (C-5'), 61.9 (C-6'), 114.4 (C-α'), 147.9 (C-β'), 101.9 (C-1''), 71.9 (C-2''), 71.8 (C-3''), 73.3 (C-4''), 70.2 (C-5''), 18.3 (C-6''), 127.3 (C-1'''), 115.2 (C-2'''), 146.8 (C-3'''), 149.9 (C-4'''), 116.5 (C-5'''), 123.2 (C-6'''), 167.6 (C=O); ESI-MS *m/z* 621.63 $[M-H]^-$ and *m/z* 623.67 $[M+H]^+$, (calcd. from $C_{29}H_{34}O_{15}$, 622.12).

Discussion of the Spectroscopic Data for Crenatoside

The ^{1}H-NMR spectrum displayed signals at δ 4.55 (1H, d, *J* = 7.8 Hz) and δ 5.15 (1H, d, *J* = 1.4 Hz) assignable to anomeric protons of two sugar moieties and at the ^{13}C-NMR spectrum the corresponding signals at δ 98.9 and 101.9 ppm were assignable to the anomeric carbons of two different sugar moieties. These chemical shifts are characteristics of the glucose and rhamnose, respectively [21, 31]. Furthermore, the value

of the coupling constant in the anomeric proton of the D-glucose (*J* = 7.8 Hz) indicated a β-configuration, while that of the L-rhamnose (*J* = 1.8 Hz) is consistent with an α-configuration [21].

The ^{1}H-NMR displayed the aromatic signals at δ 6.78; 6.97 and 7.06 ppm assigned to an ABX system that correspond to a caffeic acid. The two doublets at δ 6.28 and 7.60 ppm are assigned to α' and β' protons of the olefinic part of the caffeoyl unit, the coupling constant (*J* = 16.0 Hz) indicating their *trans* configuration [21, 31].

A second aromatic ABX system was observed with chemical shifts at δ 6.69; 6.73 and 6.82 ppm, by the protons of the 3,4-dihydroxyphenylethanol moiety. The chemical shift of the double doublet at δ 4.60 ppm assignable to the proton at the β carbon indicated that this carbon is oxygenated, which was further confirmed from the upfield shift of its ^{13}C-NMR signal at δ 78.2 ppm [21, 31].

The exact position of the sugar attachment (β-D-glucose and α-L-rhamnose), caffeic acid and 3,4-dihydroxyphenylethanol were determined by signal comparison of the ^{1}H-NMR and ^{13}C-NMR with the previously reported data described [21]. Thus, the isolated compound was established as the phenylethanoid glycoside crenatoside {1',2'-[β(3,4-dihydroxyphenyl)-α, β-dioxoethanol]-4'-*O*-caffeoyl-*O*-α-L-rhamnopyranosyl-(1→3)-*O*-β-D-glucopyranoside}.

Figure 9S – ^{1}H-NMR spectrum of crenatoside (400 MHz, DMSO-d6 e MeOD,

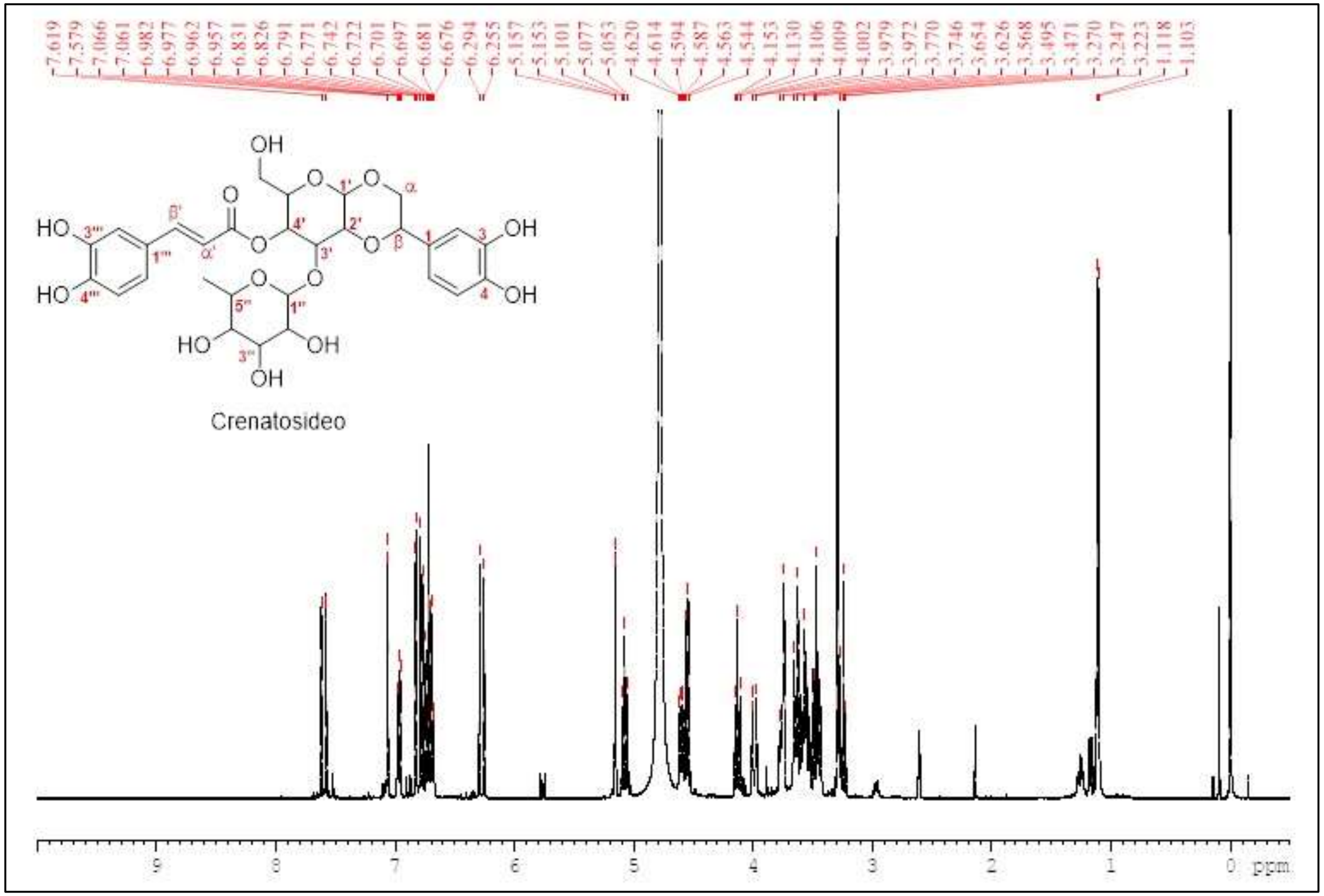

Figure 10S – ^{1}H-NMR spectrum expansion 7.7 to 6.3 ppm of crenatoside (400 MHz, DMSO-d6 e MeOD, δ)

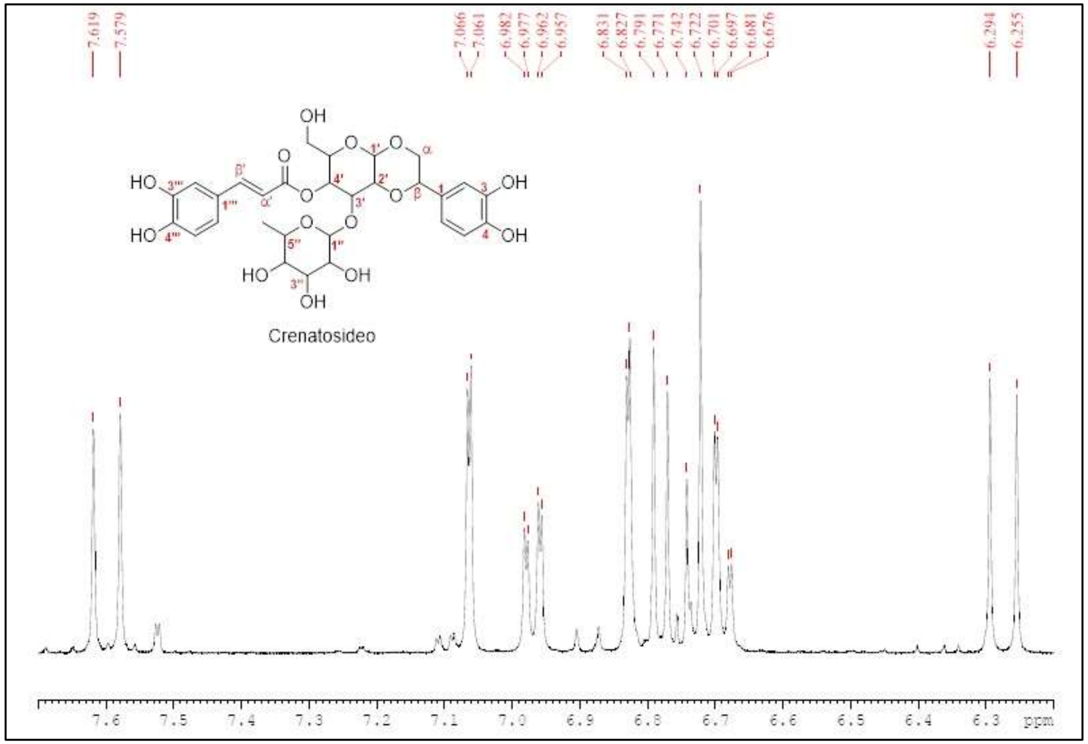

Figure 11S – ^{1}H-NMR spectrum expansion 5.2 to 4.5 ppm of crenatoside (400 MHz, DMSO-d6 e MeOD, δ)

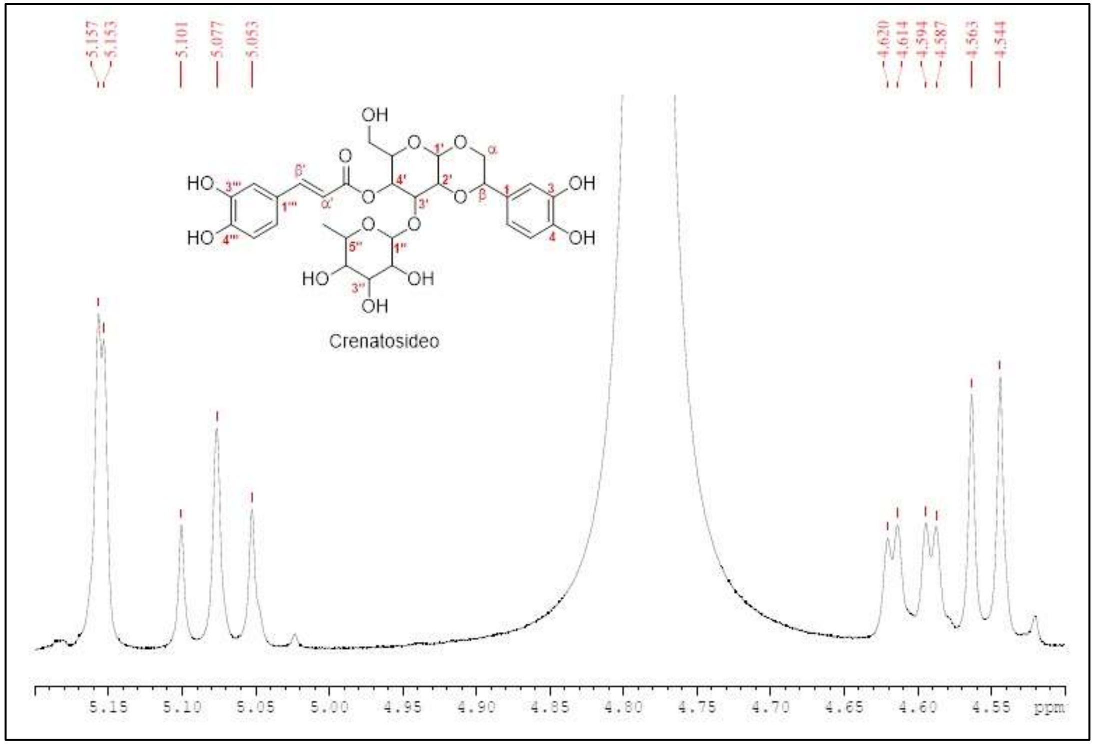

Figure 12S – ^{1}H-NMR spectrum expansion 4.2 to 3.2 ppm of crenatoside (400 MHz, DMSO-d6 e MeOD, δ)

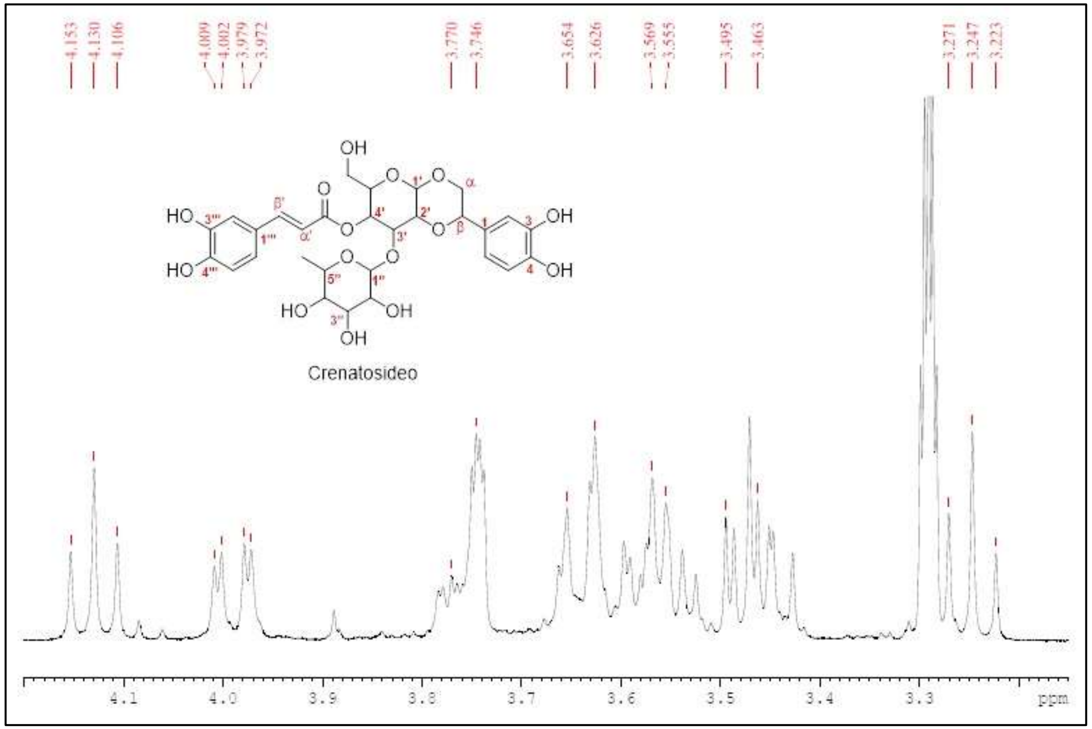

Figure 13S – ^{1}H-NMR spectrum expansion 1.3 to 0 ppm of crenatoside (400 MHz, DMSO-d6 e MeOD, δ)

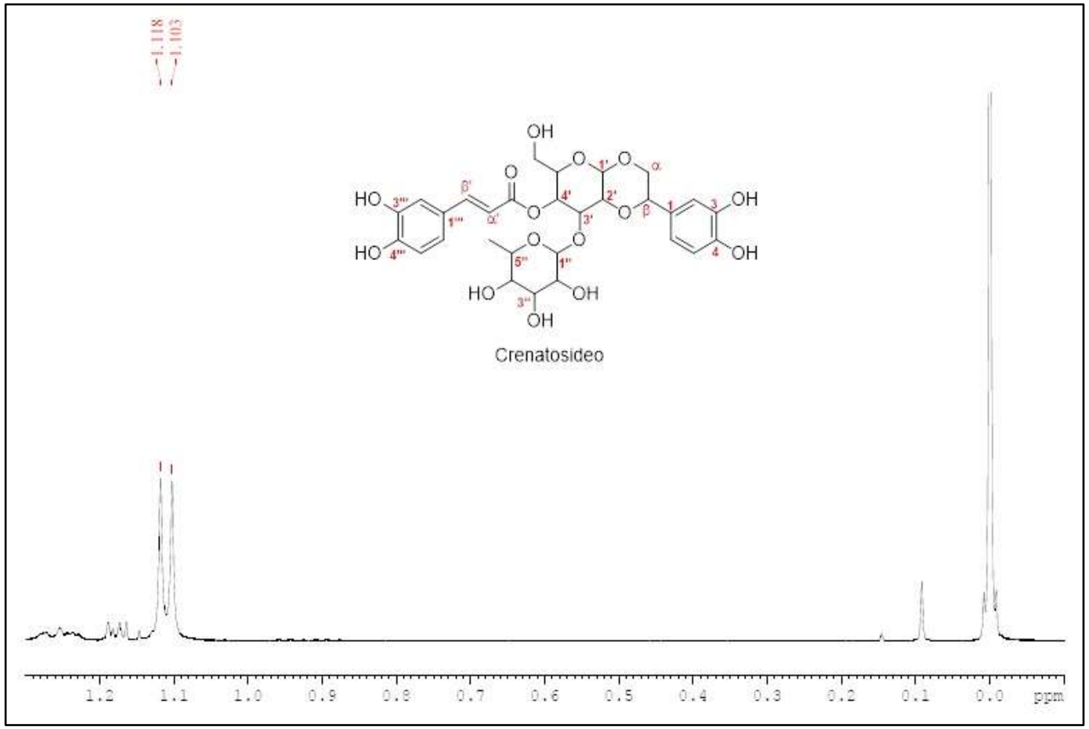

Figure 14S – ^{13}C-NMR spectrum of crenatoside (100 MHz, DMSO-d6 e MeOD, δ)

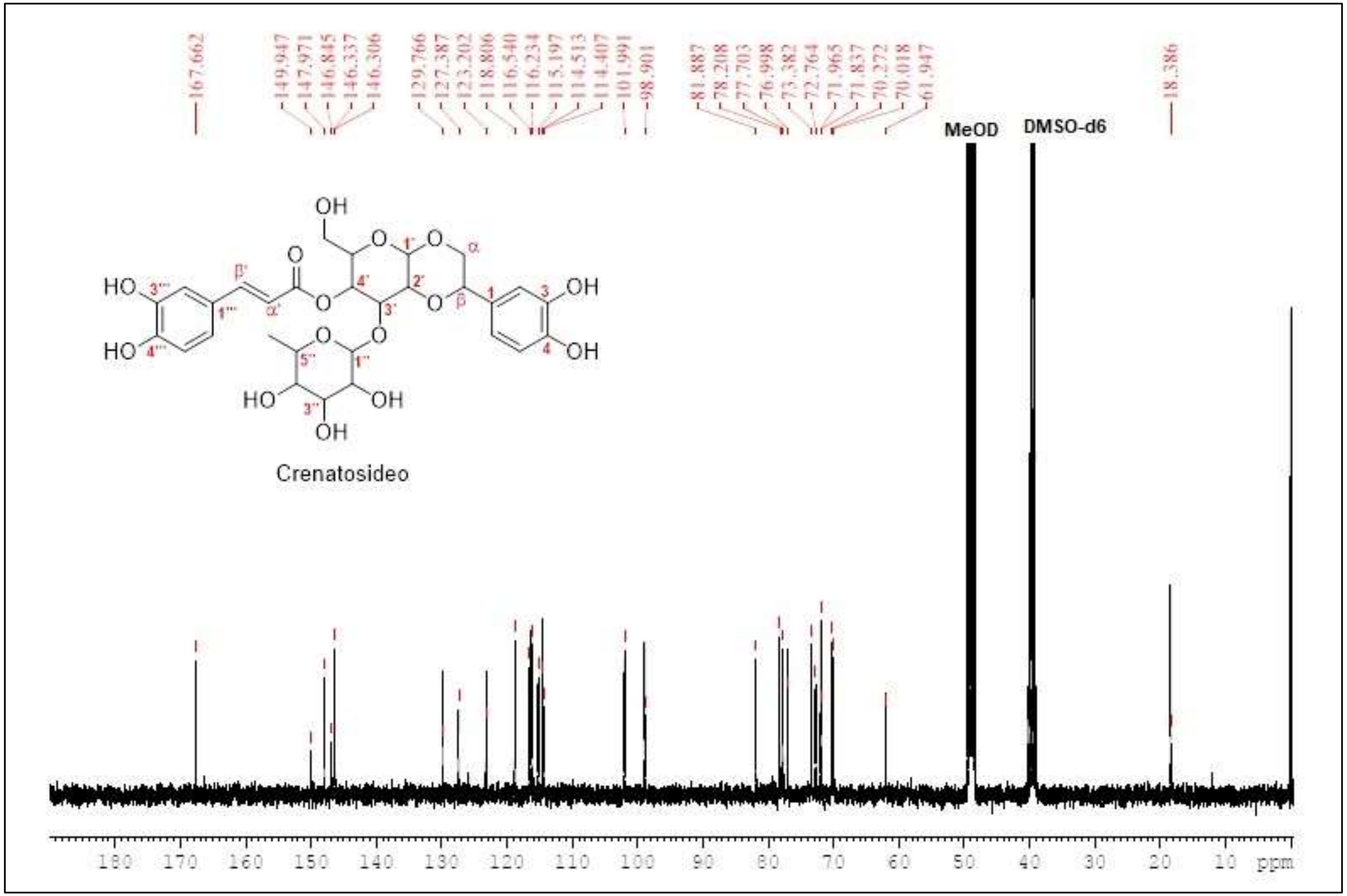

15S – ^{13}C-NMR spectrum of crenatoside (DEPT-135, 100 MHz, DMSO-d6 e MeOD, δ)

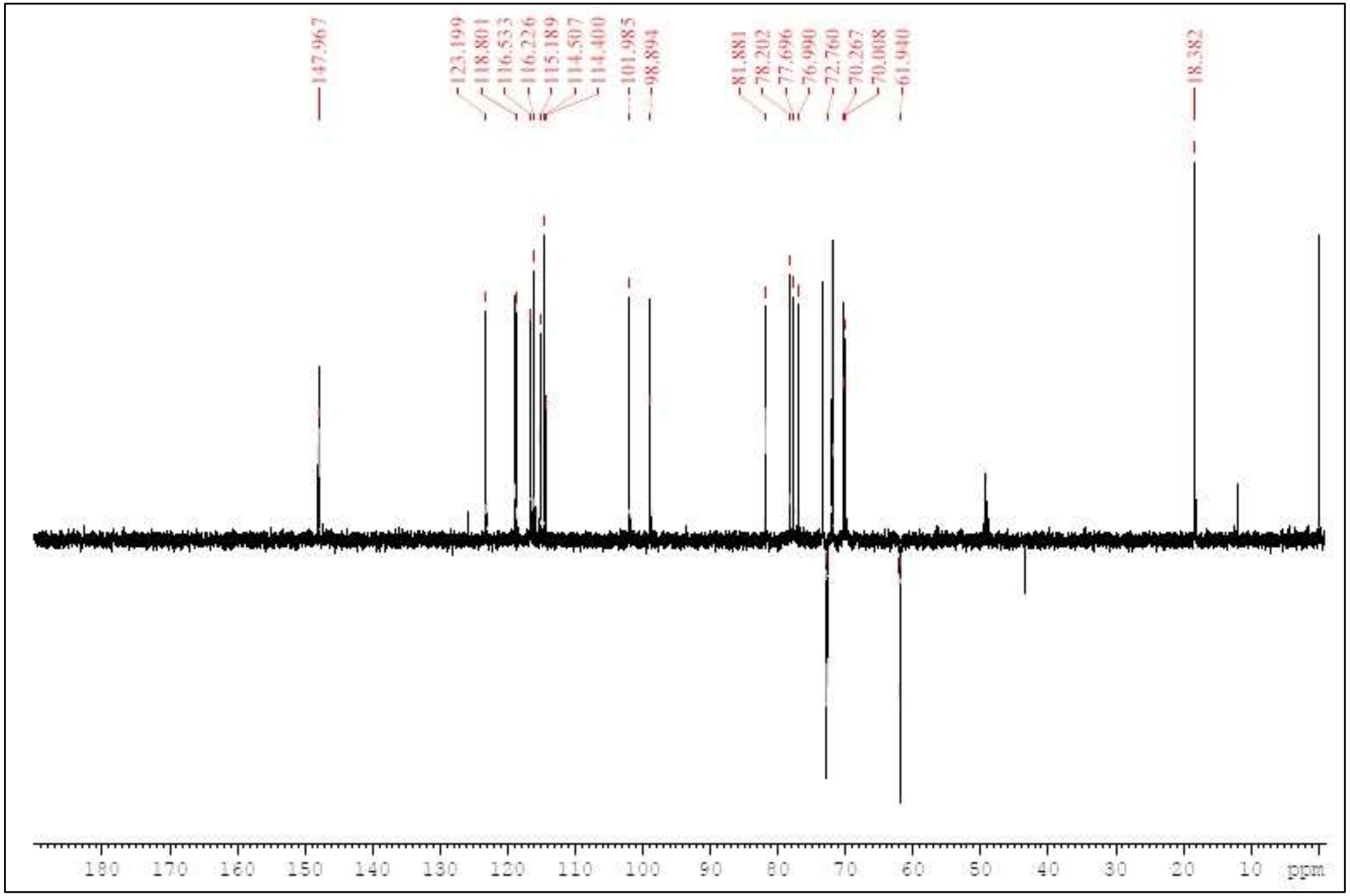
147.967
123.199
118.801
116.533
116.226
115.189
114.507
114.400
101.985
98.894
81.881
78.202
77.696
76.990
72.760
70.267
70.008
61.940
18.382
180
170
160
150
140
130
120
110
100
90
80
70
60
50
40
30
20
10
ppm
